# Assessment of recurrent fever among children undergoing tonsillectomy

**DOI:** 10.1186/s12887-024-05312-x

**Published:** 2024-12-27

**Authors:** Mana Espahbodi, Kathryn M. Edwards, Steven L. Goudy, Edward B. Penn, Kalpana Manthiram

**Affiliations:** 1 Department of Otolaryngology-Head and Neck Surgery, University of Utah School of Medicine, Salt Lake City, UT USA; 2 Vanderbilt University School of Medicine, Nashville, TN USA; 3 Division of Pediatric Infectious Diseases, Department of Pediatrics, Vanderbilt University School of Medicine, Nashville, TN USA; 4 Department of Otolaryngology-Head and Neck Surgery, Emory University School of Medicine, Atlanta, GA USA; 5 Greenville Health System Children’s Hospital, Greenville, SC USA; 6 Laboratory of Immune System Biology, National Institute of Allergy and Infectious Diseases, National Institutes of Health, Bethesda, MD USA

**Keywords:** Periodic fever, Aphthous stomatitis, Pharyngitis, And cervical adenitis syndrome; tonsillectomy; recurrent tonsillitis

## Abstract

**Background:**

Recurrent tonsillitis is a common indication for tonsillectomy in children and has phenotypic overlap with periodic fever, aphthous stomatitis, pharyngitis, and cervical adenitis (PFAPA) syndrome. We sought to characterize symptoms associated with PFAPA among children undergoing tonsillectomy.

**Methods:**

Parents/guardians of children undergoing tonsillectomy at Vanderbilt Children’s Hospital over a six-week period were queried regarding symptoms of recurrent fever. Follow-up questionnaires were administered 3 and 12 months after tonsillectomy.

**Results:**

82% (120/147) of patients who underwent tonsillectomy during the study period participated. Provider-documented indications for tonsillectomy were obstructive sleep apnea in 88% and recurrent tonsillitis in 33%. 11% (13/120) reported$\:\:\ge\:$6 episodes of stereotypical fever in a one-year period. During febrile episodes among these 13 subjects, 11 had tonsillitis, 5 had cervical adenitis, 3 had aphthous stomatitis, and three reported regular and predictable episode timing. In addition, participants with ≥3 episodes/year of recurrent febrile tonsillitis (*N* = 33) had a significantly higher prevalence of recurrent aphthous ulcers than those without recurrent tonsillitis (24% vs. 9%, *p* = 0.04). All participants, including those with recurrent fever, reported fewer febrile tonsillitis episodes one year after tonsillectomy.

**Conclusions:**

In our survey of children undergoing tonsillectomy, a subpopulation had frequent, stereotypical fever episodes with recurrent tonsillitis, aphthous stomatitis, or regular timing like patients with PFAPA. Although we cannot diagnose such patients with PFAPA in this limited retrospective study, pediatricians and otolaryngologists evaluating patients for tonsillectomy should be aware of the clinical signs of PFAPA that may warrant additional evaluation and therapeutic approaches.

**Supplementary Information:**

The online version contains supplementary material available at 10.1186/s12887-024-05312-x.

## Background

Recurrent tonsillitis, or repeated episodes of palatine tonsil inflammation, is a common indication for tonsillectomy [1]. Prospective randomized controlled trials of tonsillectomy in children with recurrent tonsillitis revealed that those with frequent episodes (≥ 7 episodes in preceding year, ≥ 5 episodes/year for 2 years, and ≥ 3 episodes/year for 3 years) meeting stringent criteria known as the Paradise Criteria had significant benefit from tonsillectomy [2]. Although children with fewer and more loosely defined episodes also had improvement post-tonsillectomy, the effect was modest in that even subjects in the non-surgical control group had very few episodes during the 3-year follow-up period [3]. The authors concluded that the risks of tonsillectomy outweighed the small benefit in patients with recurrent tonsillitis who did not meet the Paradise Criteria.

Recent guidelines for tonsillectomy in children recommend “watchful waiting” for children with recurrent tonsillitis who do not meet Paradise Criteria; however, these guidelines also recommend screening these patients for periodic fever, aphthous stomatitis, pharyngitis, and cervical adenitis (PFAPA) syndrome [1, 4]. Periodic fever syndromes are characterized by recurrent, stereotypical, noninfectious episodes of fever. Of these syndromes, PFAPA syndrome is considered to be the most common in children [4]. Children with PFAPA typically present with regularly recurring stereotypical fevers episodes accompanied by aphthous stomatitis, pharyngitis, and/or cervical adenitis, but between flares, they are asymptomatic with normal growth and development [5, 6]. In both observational studies and randomized controlled trials, tonsillectomy was shown to markedly reduce or eliminate febrile episodes in a majority of patients with PFAPA [7–9].

Little is known about the true incidence of PFAPA among children undergoing tonsillectomy in a pediatric hospital as the symptoms of PFAPA may be presumed to be due to repeated infections. A Norwegian study described the incidence of PFAPA as 2.3 per 10,000 children under 5 years of age in one region of that country [10]. In a study at a major children’s hospital in the United States, 5 children with PFAPA-like symptoms (periodic fever and aphthous stomatitis with pharyngitis) were identified among 117 (4.3%) children who underwent tonsillectomy [11]. Among 100 children undergoing tonsillectomy during the COVID-19 pandemic, 7 were reported to have a diagnosis of PFAPA at another major children’s hospital [12].

However, because of difficulties differentiating the clinical features of PFAPA from recurrent infections, PFAPA may be misdiagnosed as infectious pharyngitis or tonsillitis [13]. Thus, the objectives of our study were to better understand the frequency of features of PFAPA among children undergoing tonsillectomy at a major children’s hospital.

## Methods

Approval for this study was obtained from the Vanderbilt University Institutional Review Board (IRB number 140323). All patients undergoing tonsillectomy at Vanderbilt Children’s Hospital in Nashville, Tennessee during a 6-week interval (March 11 – April 24, 2015) were identified and informed of the study by mail. This truncated period of enrollment coincided with a medical school research elective by one of the authors (M.E.). On the day of surgery, an investigator (M.E. or K.M.) approached each patient’s parent/guardian in person to obtain informed consent and to administer a detailed structured survey on episodes of fever and pharyngitis/tonsillitis (Supplementary File). Fever was defined as a temperature greater than 38.3^o^C (101^o^F), and pharyngitis/tonsillitis was defined as a painful pharynx or reported physical examination findings of tonsillar erythema and/or exudate. Cervical adenopathy was defined as noticeable, tender neck mass(es), and aphthous stomatitis was defined as painful, shallow ulcer(s) inside the mouth. Participants were also questioned about their demographic characteristics, past medical history, family history, and symptoms. Others have defined periodic or recurrent fever as 3 or more *stereotypical* episodes of fever in a 6 month period without an alternate explanation [14–16]; similarly, we considered participants with 6 or more *stereotypical* episodes (episodes identical to one another) of fever in a 12 month period as having illness suspicious for a recurrent fever syndrome and asked further questions to understand the nature of the episodes. Investigators also extracted the indication for surgery and the physical examination from the otolaryngologist’s note in the patient record. Patient medical records from the providers who referred the patients to the otolaryngologists were not available for review. All survey responses and patient data were entered into REDCap, a web-based data capture tool [17].

At both three months and one year after tonsillectomy, parents/guardians were contacted by telephone and surveyed regarding postoperative complications and persistent symptoms after tonsillectomy.

Fisher’s exact test or Mann-Whitney U test was used to compare the features of those with and without frequent tonsillitis. The number of episodes of tonsillitis with fever before and after tonsillectomy were compared with Wilcoxon signed-rank test, a matched statistical test. All analyses were performed with SPSS version 23.

## Results

### Participant characteristics and comorbid conditions

During the 6-week enrollment period, 147 parents/guardians were approached and a total of 120 agreed to participate (82% participation rate). 90% of participants also underwent an adenoidectomy at the time of tonsillectomy. Demographic data and other patient characteristics, including pertinent past medical history, past surgical history, and indications for tonsillectomy in the enrolled patients are described in Table 1. Sleep-disordered breathing or obstructive sleep apnea (OSA) (106/120 or 88%) and recurrent tonsillitis (39/120 or 33%) were the most common indications for tonsillectomy reported by otolaryngology providers. No participants were previously diagnosed with PFAPA or referred for tonsillectomy for this indication. Nearly 28% (33/120) of participants reported at least three episodes of tonsillitis with fever per year; these participants had a significantly higher prevalence of recurrent aphthous ulcers than those with fewer than three episodes of tonsillitis with fever per year (24% vs. 9%, *p* = 0.04).

**Table 1:** Characteristics of all participants

	All subjects (*N* = 120) Mean (standard error) or Percentage
Age (years)	6.8 (0.3)
Gender	51% female 49% male
Ethnicity	90% non-Hispanic 10% Hispanic
Race*	83% White 18% African-American 13% American Indian 3% Asian 1% other 1% unknown
Past medical history*	15% developmental delay 12% speech delay 3% genetic disorder 28% asthma 52% allergic rhinitis 21% eczema 19% preterm delivery
Other otolaryngologic problems*	13% recurrent aphthous ulcers 35% recurrent otitis media 46% dysphagia 38% dysphonia 35% receiving speech therapy
Past otolaryngology surgical history*	23% tympanostomy tubes 1% prior tonsillectomy 9% prior adenoidectomy
Indications for tonsillectomy per otolaryngology providers*	88% for OSA or sleep-disordered breathing 33% for recurrent tonsillitis 13% for dysphagia 1% for dysphonia

* Percentages do not add up to 100% since more than one entity could be selected

Nearly 11% (13/120) of participants had six or more stereotypical fever episodes (all episodes identical to one another) in a 12-month period over the past three years; these patients had an average of 7.8 (± 0.7 standard error) episodes of fever in the 12 months before tonsillectomy. The characteristics of febrile episodes in those 13 patients who reported frequent recurrent febrile episodes are shown in Table 2. Three patients had aphthous ulcers during fever episodes, five had cervical adenitis, 11 had erythematous tonsillitis, and 9 had exudative tonsillitis. Three patients reported episodes that occurred so regularly that the parents/guardians could predict episode timing. In those with frequent, stereotypic fever episodes, the mean age at onset of fever episodes was 34 (± 8.5) months, fever episodes lasted 3 (± 0.3) days, and the mean interval between fevers was 36 (± 5.8) days. The mean height of fever was 102.7 (± 0.4) degrees F. Table 3 shows each participant’s symptoms. Seven subjects reported cough or rhinorrhea during flares, but 6 had neither of these viral upper respiratory tract symptoms during episodes.

**Table 2:** Clinical characteristics of patients with recurrent fever ($\:\ge\:$6 stereotypical fever episodes per year) compared to those of PFAPA patients in the literature

Characteristic	Mean (standard error) OR Number of participants with symptom (%) *N* = 13	Marshall et al. 1987 [1] *N* = 12	Thomas et al. 1999 [2] *N* = 94	Feder and Salazar 2010 [15] *N* = 105
Age of onset of fever	34 (8.5) months		33.6 months	39.6 months
Duration of fever episode	3 (0.3) days	5 days	4.8 days	4.1 days
Interval between episodes	36 (5.8) days	31.5 days	28.2 days	29.8 days
Symptoms during episodes				
Aphthous stomatitis	3 (23%)	75%	67%	38%
Cervical lymphadenopathy	5 (38%)	67%	77%	62%
Pharyngitis	11 (85%)	75%	65%	85%
Tonsillar exudate	9 (69%)			
Tonsillar erythema	11 (85%)			
Cough	6 (46%)		20%	
Rhinorrhea	6 (46%)		18%	
Headache	5 (38%)		65%	44%
Abdominal pain	4 (31%)	50%	45%	41%
Nausea and/or vomiting	6 (46%)	50%	52%	27%
Diarrhea	2 (15%)		30%	
Rash	2 (15%)		15%	
Arthralgias and/or myalgias	4 (31%)			

**Table 3:** Symptoms of the 13 patients with recurrent fever ($\:\ge\:$6 stereotypical fever episodes per year)

#	Age at onset (months)	Regular timing of episodes	Duration of fever episodes (days)	Interval between episodes (days)	Aphthous stomatitis	Pharyngitis	Cervical lymphadenopathy	Diagnosed with sleep disordered breathing
1	12	-	3.0	Unknown but 8 in the past year	-	-	-	-
2	95	-	2.0	52	-	+	-	-
3	60	-	1.0	60	+	-	-	+
4	36	-	2.0	30	+	+	-	+
5	12	-	3.0	Unknown but 6 in the past year	+	+	-	+
6	3	-	4.0	30	-	-	-	+
7	48	-	3.0	42	-	+	+	+
8	22	+	2.5	10	-	+	-	-
9	84	-	1.0	75	-	+	+	+
10	12	+	5.0	21	-	+	+	+
11	48	-	4.0	21	-	+	-	+
12	10	-	4.0	30	-	+	+	-
13	5	+	3.0	30	-	+	+	+

Twelve of the 13 patients with recurrent fever had testing for group A streptococcus (GAS) from the pharynx during episodes. Four tested positive for GAS for all episodes, seven during some but not all fever episodes, and one had no positive tests for GAS. Only two out of 13 patients with recurrent fever were diagnosed as having recurrent tonsillitis by their otolaryngology provider, but 9 of their parents/guardians referenced recurrent tonsillitis as the primary reason for tonsillectomy. Additionally, nine patients with recurrent fever had a diagnosis of sleep disordered breathing or OSA, three of whom had polysomnography confirming OSA.

### Outcomes following tonsillectomy

76% (91/120) of participants completed a follow-up survey 3 months after tonsillectomy. 8% (7/91) reported complications related to tonsillectomy that warranted an emergency room visit and/or inpatient admission after tonsillectomy. In these patients with complications, 3 had hemorrhage, 2 had both dehydration and inadequate pain control, and 1 had inadequate pain control alone. Of the 13 subjects with recurrent fever, only one had both dehydration and inadequate pain control requiring an emergency room visit after tonsillectomy.

**Fig. 1:**
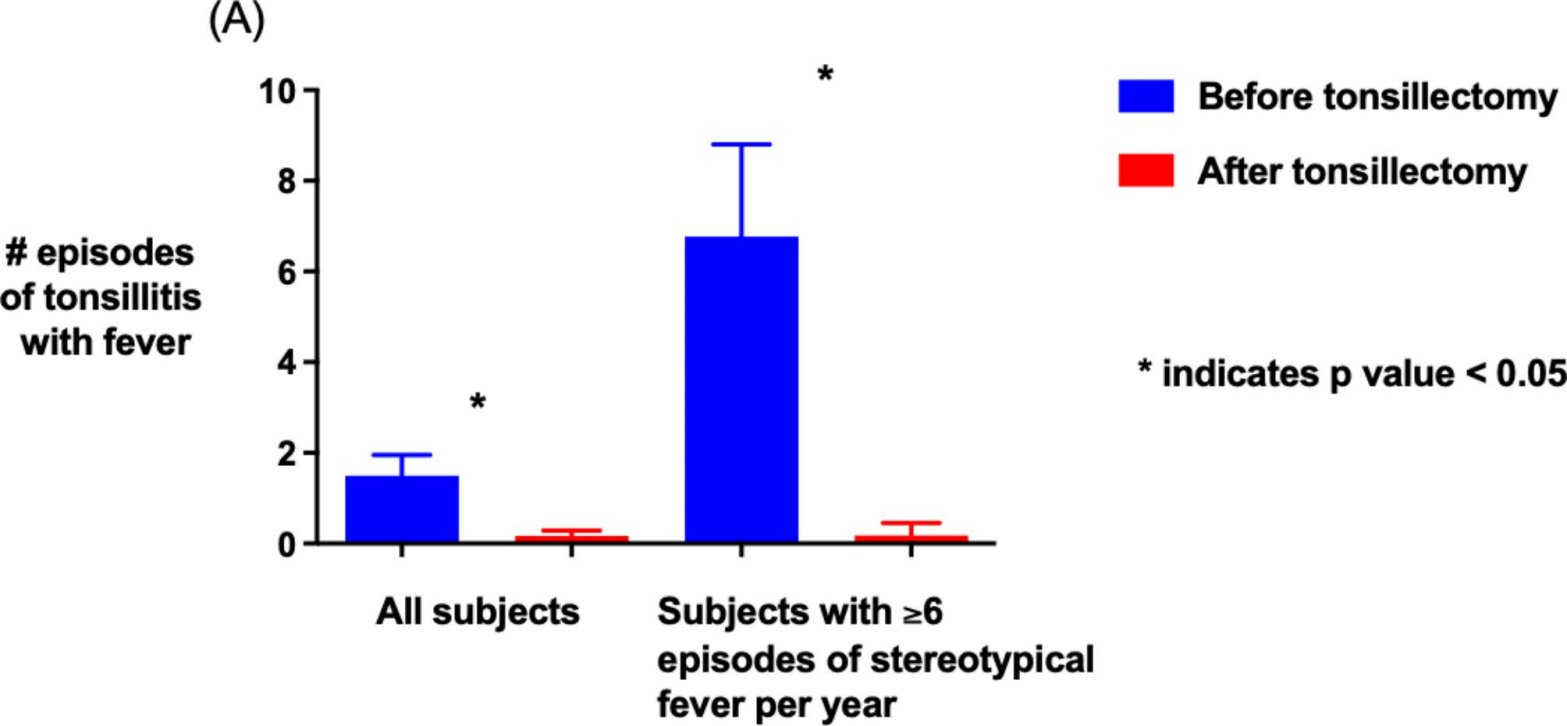
Comparison of the mean number of episodes of tonsillitis with fever one year before and after tonsillectomy in all subjects and in those recurrent fever ($\:\ge\:6\:\text{e}\text{p}\text{i}\text{s}\text{o}\text{d}\text{e}\text{s}/\text{y}\text{e}\text{a}\text{r})$. Error bars represent 95% confidence intervals

74% (89/120) of participants completed a follow-up survey one year after tonsillectomy. Among the full cohort of participants, significant declines in the number of febrile tonsillitis episodes (1.5 vs. 0.2 episodes per year before and after tonsillectomy, *p* < 0.001) were noted in the year after tonsillectomy (Fig. 1a-b).

Eleven of the 13 participants with more than 6 stereotypical fever episodes per year were followed for 1 year after tonsillectomy. These participants also had significant (*p* < 0.05) declines in the number of febrile tonsillitis episodes (Fig. 1). Nine of the 11 participants (82%) had complete resolution of fever episodes after tonsillectomy and two had only a single febrile episode with tonsillitis in the year following tonsillectomy.

## Discussion

We assessed children undergoing tonsillectomy during a short-defined interval at a major children’s hospital for features of recurrent fever and PFAPA. Our findings indicate that a subset of subjects with recurrent fever have symptoms and characteristics noted in patients with PFAPA, but often with an incomplete presentation. In particular, three of these participants had recurrent aphthous ulcers during repeated flares and another three reported regular episode timing, which are unique features of PFAPA that are rarely found in patients with recurrent infectious tonsillitis. Without access to the retrospective medical records of the children undergoing tonsillectomy from the referring providers, we were unable to determine whether these patients met the strict diagnostic criteria of PFAPA. However, the presence of PFAPA-like features raises questions about whether closer prospective analysis of their recurrent fever episodes would have resulted in the diagnosis of PFAPA in some of these patients. Even children with “incomplete PFAPA” have been reported to have a good response to tonsillectomy [18]. In addition, many participants with recurrent fever also had symptoms consistent with sleep disordered breathing or OSA, which may have drawn the focus of the provider away from evaluation of recurrent fever and PFAPA.

Testing indicating the presence GAS in the pharynx does not preclude PFAPA since over 15% of healthy, asymptomatic school-aged children are colonized with GAS in their pharynx at one point in time and around 30% may be colonized over the course of a year [19]. Among the patients we identified with recurrent fever, one patient who had repeated positive testing for GAS was only two years old and GAS pharyngitis is rare in children less than 3 years of age [20]. Another participant who tested positive for GAS often (but not during all episodes) reported aphthous stomatitis during stereotypical fever episodes; however, aphthous ulcers are not seen in GAS pharyngitis [20]. In these two participants, positive GAS testing may have indicated colonization and not true infection although GAS testing was not done during asymptomatic periods to confirm this hypothesis. Thus, in patients with recurrent fevers with features of PFAPA and positive testing for GAS, physicians should determine whether the symptoms are compatible with GAS pharyngitis, GAS colonization or PFAPA. Careful physical examination during episodes and detailed history of timing would likely identify more specific signs of PFAPA such as recurrent aphthous ulcers and regular timing of flares. Table 4 lists screening questions pediatricians and otolaryngologists may consider in their history to identify patients with features of PFAPA among those with recurrent tonsillitis.

**Table 4:** Screening questionnaire for PFAPA

1. How many episodes of fever (temperature > 100.4 F) has your child had in the past year? *More than 6 episodes in a year may raise your suspicion.*
2. Are all of these fever episodes similar to each other in terms of symptoms and duration? *Stereotypical nature of episodes in terms of symptoms and duration is critical to the diagnosis of periodic fever. URI symptoms like cough*,* rhinorrhea*,* and nasal congestion are rarely seen in PFAPA.*
3. Does your child have aphthous ulcers or canker sores during their fever episodes? *Although the absence of aphthous ulcers does not preclude the diagnosis of PFAPA*,* the presence of aphthous ulcers during flares is suggestive of PFAPA.*
4. What are the dates of the fever episodes over the last year? Do the episodes occur with regular timing? *Regular timing of fever episodes is highly suggestive of PFAPA. However*,* patients with PFAPA may skip episodes or have periods of remission.*
5. Is the child asymptomatic between fever episodes and otherwise growing and developing normally?

Interestingly, we found that children with 3 or more episodes of tonsillitis with fever per year were significantly more likely to have a history of recurrent aphthous ulcers than children undergoing tonsillectomy for other reasons. We previously found that first-degree family members of patients with PFAPA have high rates of recurrent aphthous ulcers suggesting a link in the pathogenesis of these oropharyngeal disorders [13]. Indeed, recently, patients with PFAPA, recurrent aphthous ulcers, and Behçet’s disease were found to have common genetic susceptibility loci, further supporting the biologic connection between aphthous ulcers and PFAPA [21]. Here, we find that aphthous ulcers are more common among children with recurrent tonsillitis, which has also noted to be heritable in studies of twins [22]. The raises the question as to whether a wider group of children with recurrent tonsillitis have inflammatory tonsillitis with genetic underpinnings similar to PFAPA. Assessment of genetic risk variants for recurrent tonsillitis may shed light on whether recurrent tonsillitis has a genetic basis and shares genetic susceptibility loci near genes involved in inflammatory immunologic pathways with PFAPA.

One year after tonsillectomy, all patients, including those with recurrent fever, experienced a decrease in the number of episodes of febrile tonsillitis, which is concordant with outcomes from other studies on recurrent tonsillitis [2, 3]. It is likely that some patients with recurrent fever in this and other reported studies may actually have PFAPA. If they had been recognized as such, could unnecessary antibiotic courses have been avoided? This question cannot be answered by this study but would be helpful to assess prospectively.

Our study has several limitations. One limitation is the potential for recall bias of parents and guardians completing the survey as we asked them to remember medical encounters and illnesses over the past several years. Additionally, symptom assessment in this study was primarily based on history. To improve the specificity of the clinical diagnoses of periodic fever syndromes, patients with a history suspicious for PFAPA should be evaluated during episodes (including detailed physical exam), have detailed prospective assessment of episode dates using fever diaries, and should have compatible laboratory testing showing elevated inflammatory markers during acute febrile episodes and normalization of markers during afebrile periods. The nature of our study made it impossible to perform such evaluations and we had to rely on parental recall. In addition, we did not review retrospective medical records from the referring physicians. The other limitation of our study was the truncated enrollment period that coincided with a medical school research elective and reflected a single time frame in only one season of the year. Finally, we also did not obtain GAS throat cultures during asymptomatic periods or do extensive testing for viral respiratory pathogens to identify those with recurrent viral pharyngitis. No samples to detect infectious agents were performed at the time of the surgery.

Despite these limitations, we identified many children with recurrent fever with features of PFAPA among those undergoing tonsillectomy. We recommend that pediatricians query parents about symptoms of PFAPA in patients with recurrent tonsillitis and recurrent fever and we recommend that otolaryngologists assess for PFAPA during preoperative evaluation for tonsillectomy. We support further prospective studies on PFAPA to determine its prevalence among children undergoing tonsillectomy and in the general population. We also support additional studies to evaluate whether children with more broadly defined “recurrent tonsillitis” shares genetic susceptibility loci with PFAPA and recurrent aphthous ulcers.

## Conclusions

Many children with recurrent fever undergoing tonsillectomy have features of PFAPA. In patients with frequent stereotypical fevers, pediatricians, and otolaryngologists should elicit a careful history to dissect the timing and duration of symptoms and perform a careful physical examination during episodes to identify those with PFAPA.

## Electronic supplementary material

Below is the link to the electronic supplementary material.

Supplementary Material 1

## Data Availability

The datasets used and/or analyzed during the current study available from the corresponding author on reasonable request.
