# Supplementary material for: Assessment of recurrent fever among children undergoing tonsillectomy
Source: BMC Pediatr. 2024 Dec 27;24:835. doi: 10.1186/s12887-024-05312-x (PMC11673685; doi:10.1186/s12887-024-05312-x)
Supplement: Supplementary file 1 — Supplementary Material 1 [file 12887_2024_5312_MOESM1_ESM.pdf]

Tonsillectomy Survey

Codebook

Data Dictionary Codebook

06/20/2019 12:49pm

Collapse all instruments

#	Variable / Field Name	Field Label<br>*Field Note*	Field Attributes (Field Type, Validation, Choices, Calculations, etc.)
Instrument: **Initial Survey** (initial_survey)			Collapse
1	record_id	Record ID	text
2	mrn	Section Header: *Identifying Information*<br>MRN	text (number), Required, Identifier
3	birthday	What is your child's birthdate?	text (date_mdy), Required, Identifier
4	gender	What is your child's gender?	radio, Required<br>1 Female<br>2 Male
5	ethnicity	What is your child's ethnicity?	radio<br>1 Hispanic<br>2 Non-hispanic
6	race	What is your child's race?	checkbox<br>Indian — race___indian — American Indian or Alaska Native<br>Islander — race___islander — Native Hawaiian or Other Pacific Islander<br>White — race___white — White (Caucasian)<br>Asian — race___asian — Asian<br>Black — race___black — Black or African American<br>Other — race___other — Other<br>Unknown — race___unknown — Unknown
7	main_ind_par	Section Header: *Basic Information and History*<br>What is the main reason why your child is getting a tonsillectomy?	radio<br>1 OSA/sleep disordered breathing<br>2 PFAPA<br>3 Recurrent tonsillitis<br>4 Dysphagia (trouble swallowing)<br>5 Dysphonia (trouble speaking)<br>6 Other<br>7 Don't know
8	oth_ind_par<br>Show the field ONLY if:<br>[main_ind_par] = '6'	Please describe.	text

9	second_ind_par	What are other reasons your child is getting their tonsils out? Check all that apply.	checkbox<br>1 second_ind_par___1 OSA/sleep disordered breathing<br>2 second_ind_par___2 PFAPA<br>3 second_ind_par___3 Recurrent tonsillitis<br>4 second_ind_par___4 Dysphagia (trouble swallowing)<br>5 second_ind_par___5 Dysphonia (trouble speaking)<br>6 second_ind_par___6 Other<br>7 second_ind_par___7 Don't know
10	oth_sec_ind_par<br>Show the field ONLY if: [second_ind_par(6)] = '1'	Please describe.	text
11	pmh	What other medical problems does your child have?	checkbox<br>1 pmh___1 Developmental delay<br>2 pmh___2 Genetic disorder<br>3 pmh___3 Craniofacial disorder<br>4 pmh___4 Asthma<br>5 pmh___5 Allergic rhinitis<br>6 pmh___6 Eczema<br>7 pmh___7 Other<br>8 pmh___8 Otherwise healthy
12	info_pmh<br>Show the field ONLY if: [pmh(1)] = '1' or [pmh(2)] = '1' or [pmh(3)] = '1' or [pmh(4)] = '1' or [pmh(5)] = '1' or [pmh (6)] = '1' or [pmh(7)] = '1'	Please provide more detail about your child's medical problems.	notes
13	meds	What medications does your child take?	checkbox<br>1 meds___1 Inhaled steroids<br>2 meds___2 Other<br>3 meds___3 No medications
14	oth_meds<br>Show the field ONLY if: [meds(1)] = '1' or [meds(2)] = '1'	Please list the medications that your child takes and how frequently they take it.	notes
15	psh	What surgeries has your child had in the past? Please check all that apply.	checkbox<br>1 psh___1 Myringotomy tubes<br>2 psh___2 Prior tonsillectomy<br>3 psh___3 Prior adenoidectomy<br>4 psh___4 Other
16	oth_psh<br>Show the field ONLY if: [psh(4)] = '1'	Please list any other surgeries that your child has had.	notes
17	gest_age	Was your child born at term or premature (before 37 weeks gestation)?	radio<br>1 Term<br>2 Pre-term<br>3 Don't know

18	daycare	Does your child attend daycare?	yesno<br>1 Yes<br>0 No
19	smoking	Does anyone who lives in the same house as your child smoke?	yesno<br>1 Yes<br>0 No
20	pfapa	Section Header: *PFAPA*<br>Has your child ever been diagnosed with the periodic fever syndrome, PFAPA?	yesno<br>1 Yes<br>0 No
21	who_diag_pfapa<br>Show the field ONLY if: [pfapa] = '1'	Who diagnosed your child with PFAPA?	text
22	r_fev	Has your child ever had 5 or more episodes of fever in one year that were very similar to each other? We define fever as temperature more than 101F.	yesno<br>1 Yes<br>0 No
23	r_ton_pha	Has your child ever had 5 or more episodes of tonsillitis or pharyngitis with fever in a year? We say fever is temperature greater than 101F.	yesno<br>1 Yes<br>0 No
24	ulcers_lad	Has your child ever had 5 or more episodes of mouth ulcers or swollen glands in the neck with fever in a year? We say fever is more than 101F.	yesno<br>1 Yes<br>0 No
25	age_first_fev<br>Show the field ONLY if: [ulcers_lad] = '1' or [r_ton_pha] = '1' or [r_fev] = '1' or [pfapa] = '1'	How old was your child (in months) when he/she had his/her first fever episode?	text (number)
26	fev_sim<br>Show the field ONLY if: [ulcers_lad] = '1' or [r_ton_pha] = '1' or [r_fev] = '1' or [pfapa] = '1'	Do all of the fever episodes seem similar to one another in terms of the symptoms your child has?	radio<br>1 Yes<br>0 No<br>2 Don't know
27	fev_temp<br>Show the field ONLY if: [ulcers_lad] = '1' or [r_ton_pha] = '1' or [r_fev] = '1' or [pfapa] = '1'	How high is the fever typically during an episode (in degrees F)?	text (number)
28	fev_days<br>Show the field ONLY if: [ulcers_lad] = '1' or [r_ton_pha] = '1' or [r_fev] = '1' or [pfapa] = '1'	What is the usual length of the fever episode (in days)?	text (number)
29	fev_days_sh<br>Show the field ONLY if: [ulcers_lad] = '1' or [r_ton_pha] = '1' or [r_fev] = '1' or [pfapa] = '1'	What is the shortest length of the fever episode (in days)?	text (number)
30	fev_days_lo<br>Show the field ONLY if: [ulcers_lad] = '1' or [r_ton_pha] = '1' or [r_fev] = '1' or [pfapa] = '1'	What is the longest length of the fever episode (in days)?	text (number)

31	inter_fev<br>Show the field ONLY if: [ulcers_lad] = '1' or [r_ton_pha] = '1' or [r_fev] = '1' or [pfapa] = '1'	Usually, how many days were there between the fever episodes?<br>Please count the number of days between the first day of one episode and the first day of fever of the next episode.	text (number)
32	inter_fev_sh<br>Show the field ONLY if: [ulcers_lad] = '1' or [r_ton_pha] = '1' or [r_fev] = '1' or [pfapa] = '1'	What is the shortest number of days between fever episodes?	text (number)
33	inter_fev_lo<br>Show the field ONLY if: [ulcers_lad] = '1' or [r_ton_pha] = '1' or [r_fev] = '1' or [pfapa] = '1'	What is the longest number of days between fever episodes?	text (number)
34	inter_fev_same<br>Show the field ONLY if: [ulcers_lad] = '1' or [r_ton_pha] = '1' or [r_fev] = '1' or [pfapa] = '1'	Is the time between episodes always about the same?	radio<br>1 Yes<br>0 No<br>2 Do not Know
35	inter_fev_reg<br>Show the field ONLY if: [ulcers_lad] = '1' or [r_ton_pha] = '1' or [r_fev] = '1' or [pfapa] = '1'	Do episodes occur so regularly that you can predict when the next one will come?	radio<br>1 Yes<br>0 No<br>2 Do not Know
36	fev_1y<br>Show the field ONLY if: [ulcers_lad] = '1' or [r_ton_pha] = '1' or [r_fev] = '1' or [pfapa] = '1'	In the last 12 months, how many episodes did your child have?	text
37	fev_1y_2ya<br>Show the field ONLY if: [ulcers_lad] = '1' or [r_ton_pha] = '1' or [r_fev] = '1' or [pfapa] = '1'	In the 12 months before that (2 years ago), how many episodes did your child have?	text
38	fev_1y_3ya<br>Show the field ONLY if: [ulcers_lad] = '1' or [r_ton_pha] = '1' or [r_fev] = '1' or [pfapa] = '1'	In the 12 months before that (3 years ago), how many episodes did your child have?	text
39	rec_pfapa<br>Show the field ONLY if: [ulcers_lad] = '1' or [r_ton_pha] = '1' or [r_fev] = '1' or [pfapa] = '1'	When did your child's most recent episode start?	text

40	fev_as_symp<br>Show the field ONLY if:<br>[ulcers_lad] = '1' or [r_ton_pha] = '1' or [r_fev] = '1' or [pfapa] = '1'	What symptoms does your child have during a fever episode?	checkbox<br>1 fev_as_symp___1 Mouth ulcer or canker sores<br>2 fev_as_symp___2 Sore throat<br>3 fev_as_symp___3 Swollen nodes/glands in the neck<br>4 fev_as_symp___4 Pus/exudate/white spots on tonsil<br>5 fev_as_symp___5 Redness (erythema) of tonsils<br>6 fev_as_symp___6 Cough<br>7 fev_as_symp___7 Runny nose<br>8 fev_as_symp___8 Headache<br>9 fev_as_symp___9 Belly pain (abdominal pain)<br>10 fev_as_symp___10 Muscle pain (myalgia) or joint pains<br>11 fev_as_symp___11 Vomiting<br>12 fev_as_symp___12 Diarrhea<br>13 fev_as_symp___13 Rash<br>14 fev_as_symp___14 Droopy or glossy eyes<br>15 fev_as_symp___15 Other
41	oth_fev_as_symp<br>Show the field ONLY if:<br>[fev_as_symp(15)] = '1'	Please describe.	notes
42	fev_ton<br>Show the field ONLY if:<br>[ulcers_lad] = '1' or [r_ton_pha] = '1' or [r_fev] = '1' or [pfapa] = '1'	Does your child have sore throat or tonsillitis during every fever episode?	yesno<br>1 Yes<br>0 No
43	fev_ton_no_exp<br>Show the field ONLY if:<br>[fev_ton] = '0'	Please explain.	notes
44	sym_bt_fev<br>Show the field ONLY if:<br>[ulcers_lad] = '1' or [r_ton_pha] = '1' or [r_fev] = '1' or [pfapa] = '1'	Does your child have symptoms between episodes?	yesno<br>1 Yes<br>0 No
45	sym_bt_fev_des<br>Show the field ONLY if:<br>[sym_bt_fev] = '1'	Please describe.	notes
46	strep_fev<br>Show the field ONLY if:<br>[ulcers_lad] = '1' or [r_ton_pha] = '1' or [r_fev] = '1' or [pfapa] = '1'	Has your child ever been tested for strep during an episode?	yesno<br>1 Yes<br>0 No
47	strep_num_1y<br>Show the field ONLY if:<br>[strep_fev] = '1'	How many times was the strep test positive in the last 12 months?	text
48	strep_num_1y_2ya<br>Show the field ONLY if:<br>[strep_fev] = '1'	How many times was the strep test positive in the 12 months before that (2 years ago)?	text
49	strep_num_1y_3ya<br>Show the field ONLY if:<br>[strep_fev] = '1'	How many times was the strep test positive in the 12 months before that (3 years ago)?	text

50	abx_tx_fev<br>Show the field ONLY if: [ulcers_lad] = '1' or [r_ton_pha] = '1' or [r_fev] = '1' or [pfapa] = '1'	Has your child ever received antibiotics for these episodes?	radio<br>1 Yes<br>0 No<br>2 Do not Know
51	abx_tx_fev_sh<br>Show the field ONLY if: [abx_tx_fev] = '1'	Do antibiotics make the episodes shorter?	radio<br>1 Yes<br>0 No<br>2 Do not Know
52	ster_tx_fev<br>Show the field ONLY if: [ulcers_lad] = '1' or [r_ton_pha] = '1' or [r_fev] = '1' or [pfapa] = '1'	Has your child ever received steroids for these episodes?	radio<br>1 Yes<br>0 No<br>2 Do not Know
53	ster_tx_fev_sh<br>Show the field ONLY if: [ster_tx_fev] = '1'	Do steroids make the episodes shorter?	radio<br>1 Yes<br>0 No<br>2 Do not Know
54	ton_lad_wpfapasym_wofev<br>Show the field ONLY if: [ulcers_lad] = '1' or [r_ton_pha] = '1' or [r_fev] = '1' or [pfapa] = '1'	Has your child had a throat or tonsil infection without fever?	radio<br>1 Yes<br>0 No<br>2 Do not Know
55	ton_lad_wpfapasym_1y<br>Show the field ONLY if: [ton_lad_wpfapasym_wofev] = '1'	How many times in the last 12 months?	text
56	ton_lad_wpfapasym_1y_2ya<br>Show the field ONLY if: [ton_lad_wpfapasym_wofev] = '1'	How many times in the the 12 months before that (2 years ago)?	text
57	ton_lad_wpfapasym_1y_3ya<br>Show the field ONLY if: [ton_lad_wpfapasym_wofev] = '1'	How many times in the the 12 months before that (3 years ago)?	text
58	add_pfapa<br>Show the field ONLY if: [ulcers_lad] = '1' or [r_ton_pha] = '1' or [r_fev] = '1' or [pfapa] = '1'	Please provide any additional information about episodes.	notes
59	ton_num_1y<br>Show the field ONLY if: [pfapa] = '0' and [r_fev] = '0' and [r_ton_pha] = '0' and [ulcers_lad] = '0'	Section Header: *Recurrent tonsillitis*<br>In the last 12 months, how many throat or tonsil infections has your child had?	text (integer)
60	ton_wfev<br>Show the field ONLY if: [ton_num_1y] >= 1	For how many of these episodes did your child have fever of 101 degrees F or higher?	text
61	ton_wstrep_pos<br>Show the field ONLY if: [ton_num_1y] >= 1	For how many of these episodes did your child have positive testing for strep?	text
62	rec_ton<br>Show the field ONLY if: [ton_num_1y] >= 1	When was the most recent episode? Please provide the date if known, otherwise estimate.	text

63	ton_num_1y_2ya<br>Show the field ONLY if:<br>[pfapa] = '0' and [r_fev] = '0' and [r_ton_pha] = '0' and [ulcers_lad] = '0'	In the 12 months before that (2 years ago), how many throat or tonsil infections did your child have?	text (integer)
64	ton_numwfev_1y_2ya<br>Show the field ONLY if:<br>[ton_num_1y_2ya] >= 1	For how many of these episodes did your child have fever of 101 degrees F or higher?	text
65	ton_numwstrep_1y_2ya<br>Show the field ONLY if:<br>[ton_num_1y_2ya] >= 1	For how many of these episodes did your child have positive testing for strep?	text
66	ton_num_1y_3ya<br>Show the field ONLY if:<br>[pfapa] = '0' and [r_fev] = '0' and [r_ton_pha] = '0' and [ulcers_lad] = '0'	In the 12 months before that (3 years ago), how many throat or tonsil infections did your child have?	text (integer)
67	ton_numwfev_1y_3ya<br>Show the field ONLY if:<br>[ton_num_1y_3ya] >= 1	For how many of these episodes did your child have fever of 101 degrees F or higher?	text
68	ton_numwstrep_1y_3ya<br>Show the field ONLY if:<br>[ton_num_1y_3ya] >= 1	For how many of these episodes did your child have positive testing for strep?	text
69	ton_as_sym<br>Show the field ONLY if:<br>[ton_num_1y] >= 1 or [ton_num_1y_2ya] >= 1 or [ton_num_1y_3ya] >= 1	What symptoms did your child have when he/she had a throat or tonsil infection? Check all that apply:	checkbox<br>1 ton_as_sym___1 Sore throat<br>2 ton_as_sym___2 Swollen lymph nodes in neck<br>3 ton_as_sym___3 Mouth ulcers<br>4 ton_as_sym___4 Red (erythema) tonsils<br>5 ton_as_sym___5 Pus/exudate/white spots on tonsils<br>6 ton_as_sym___6 Headache<br>7 ton_as_sym___7 Abdominal pain (belly pain)<br>8 ton_as_sym___8 Muscle aches (myalgia)<br>9 ton_as_sym___9 Other
70	oth_ton_as_sym<br>Show the field ONLY if:<br>[ton_as_sym(9)] = '1'	Please describe "other"	text
71	add_ton<br>Show the field ONLY if:<br>[ton_num_1y] >= 1 or [ton_num_1y_2ya] >= 1 or [ton_num_1y_3ya] >= 1	Please provide any additional information on tonsillitis episodes.	notes
72	psq1	Section Header: *Pediatric Sleep Questionnaire*<br>When sleeping, does your child snore more than half the time?	radio<br>1 Yes<br>0 No<br>2 Don't know
73	psq2	When sleeping, does your child always snore?	radio<br>1 Yes<br>0 No<br>2 Don't know
74	psq3	When sleeping, does your child snore loudly?	radio<br>1 Yes<br>0 No<br>2 Don't know

75	psq4	When sleeping, does your child have "heavy" or loud breathing?	radio<br>1 Yes<br>0 No<br>2 Don't know
76	psq5	When sleeping, does your child have trouble breathing, or struggle to breathe?	radio<br>1 Yes<br>0 No<br>2 Don't know
77	psq6	Have you ever seen your child stop breathing during the night?	radio<br>1 Yes<br>0 No<br>2 Don't know
78	psq7	Does your child tend to breathe through the mouth during the day?	radio<br>1 Yes<br>0 No<br>2 Don't know
79	psq8	Does your child have a dry mouth on waking up in the morning?	radio<br>1 Yes<br>0 No<br>2 Don't know
80	psq9	Does your child occasionally wet the bed?	radio<br>1 Yes<br>0 No<br>2 Don't know
81	psq10	Does your child wake up feeling unrefreshed in the morning?	radio<br>1 Yes<br>0 No<br>2 Don't know
82	psq11	Does your child have a problem with sleepiness during the day?	radio<br>1 Yes<br>0 No<br>2 Don't know
83	psq12	Has a teacher or other supervisor commented that your child appears sleepy during the day?	radio<br>1 Yes<br>0 No<br>2 Don't know
84	psq13	Is it hard to wake your child up in the morning?	radio<br>1 Yes<br>0 No<br>2 Don't know
85	psq14	Does your child wake up with headaches in the morning?	radio<br>1 Yes<br>0 No<br>2 Don't know
86	psq15	Did your child stop growing at a normal rate at any time since birth?	radio<br>1 Yes<br>0 No<br>2 Don't know

87	psq16	Is your child overweight?	radio<br>1 Yes<br>0 No<br>2 Don't know
88	psq17	Does your child often not seem to listen when spoken to directly?	radio<br>1 Yes<br>0 No<br>2 Don't know
89	psq18	Does your child often have difficulty organizing tasks and activities?	radio<br>1 Yes<br>0 No<br>2 Don't know
90	psq19	Is your child often easily distracted by extraneous stimuli?	radio<br>1 Yes<br>0 No<br>2 Don't know
91	psq20	Does your child fidget with his/her hands or feet or squirms in his/her seat?	radio<br>1 Yes<br>0 No<br>2 Don't know
92	psq21	Is your child 'on the go' or often act as if 'driven by a motor'?	radio<br>1 Yes<br>0 No<br>2 Don't know
93	psq22	Does your child often interrupt or intrude on others (e.g. butts into conversations or games)?	radio<br>1 Yes<br>0 No<br>2 Don't know
94	ton_wabscess	Section Header: *Other Symptoms*<br>Has your child ever had an abscess in their tonsils?	yesno<br>1 Yes<br>0 No
95	date_ton_wabscess<br>Show the field ONLY if:<br>[ton_wabscess] = '1'	When? Please provide the date if known, otherwise estimate.	text
96	ton_wabscess_side<br>Show the field ONLY if:<br>[ton_wabscess] = '1'	Which side?	radio<br>left Left<br>right Right<br>bilateral Both<br>unknown Do not know
97	ton_wabscess_num<br>Show the field ONLY if:<br>[ton_wabscess] = '1'	How many times?	text (integer)
98	ton_wabscess_tx<br>Show the field ONLY if:<br>[ton_wabscess] = '1'	How was it treated?	radio<br>1 Antibiotics<br>2 Surgery<br>3 Other<br>4 Don't know

99	ton_wabscess_tx_oth<br>Show the field ONLY if:<br>[ton_wabscess_tx] = '3'	Please describe.	notes
100	canker_sores_or_ulcers	Has your child had canker sores or ulcers in his/her mouth more than once?	radio<br>1 Yes<br>0 No<br>2 Don't know
101	ear_infxn	What is the most number of ear infections your child has had in one year?	text
102	max_ear_infxn_12m<br>Show the field ONLY if:<br>[ear_infxn] >= 1	How many ear infections has your child had in the last 12 months?	text
103	ear_infxn_6m<br>Show the field ONLY if:<br>[ear_infxn] >= 1	How many ear infections has your child had in the past 6 months?	text
104	dysphagia	Does your child ever have difficulty swallowing?	yesno<br>1 Yes<br>0 No
105	dysphagia_as_sym<br>Show the field ONLY if:<br>[dysphagia] = '1'	Is your child's difficulty swallowing related to any of the following symptoms? Please check all that apply.	checkbox<br>1 dysphagia_as_sym___1 Fever<br>2 dysphagia_as_sym___2 Tonsillitis<br>3 dysphagia_as_sym___3 Aphthous ulcers<br>4 dysphagia_as_sym___4 Otitis media<br>5 dysphagia_as_sym___5 Large tonsils<br>6 dysphagia_as_sym___6 Don't know<br>7 dysphagia_as_sym___7 Other
106	dysphagia_as_sym_oth<br>Show the field ONLY if:<br>[dysphagia_as_sym(7)]='1'	Describe other.	text
107	dysphonia	Does your child ever have difficult speaking?	yesno<br>1 Yes<br>0 No
108	dysphonia_as_sym<br>Show the field ONLY if:<br>[dysphonia] = '1'	Is your child's difficulty speaking related to any of the following symptoms? Please check all that apply.	checkbox<br>1 dysphonia_as_sym___1 Fever<br>2 dysphonia_as_sym___2 Tonsillitis<br>3 dysphonia_as_sym___3 Aphthous ulcers<br>4 dysphonia_as_sym___4 Otitis media<br>5 dysphonia_as_sym___5 Large tonsils<br>6 dysphonia_as_sym___6 Don't know<br>7 dysphonia_as_sym___7 Other
109	dysphonia_as_sym_oth<br>Show the field ONLY if:<br>[dysphonia_as_sym(7)]='1'	Describe other.	text
110	speech_tx	Has your child ever received speech therapy?	yesno<br>1 Yes<br>0 No
111	sdays_miss	How many days of school has your child missed in the last school year?	text (integer)
112	sdays_miss_ton	How many of these missed school days were due to tonsil problems?	text

113	num_abx	How many times did your child get a course of antibiotics in the last year?	text
114	mom_age	Section Header: *Family History*<br>How old is your child's mother?	text
115	mom_medprob	Does your child's mother have any of the following conditions?	checkbox<br>1 | mom_medprob___1 | Recurrent tonsillitis<br>2 | mom_medprob___2 | PFAPA<br>3 | mom_medprob___3 | OSA diagnosed as a child<br>4 | mom_medprob___4 | OSA diagnosed as an adult<br>5 | mom_medprob___5 | Tonsillectomy<br>6 | mom_medprob___6 | Recurrent otitis media or ear infections<br>7 | mom_medprob___7 | Myringotomy tubes or ear tubes<br>8 | mom_medprob___8 | Recurrent aphthous ulcers or canker sores<br>9 | mom_medprob___9 | None of the above
116	father_age	How old is your child's father?	text
117	dad_medprob	Does your child's father have any of the following conditions?	checkbox<br>1 | dad_medprob___1 | Recurrent tonsillitis<br>2 | dad_medprob___2 | PFAPA<br>3 | dad_medprob___3 | OSA diagnosed as a child<br>4 | dad_medprob___4 | OSA diagnosed as an adult<br>5 | dad_medprob___5 | Tonsillectomy<br>6 | dad_medprob___6 | Recurrent otitis media or ear infections<br>7 | dad_medprob___7 | Myringotomy tubes or ear tubes<br>8 | dad_medprob___8 | Recurrent aphthous ulcers or canker sores<br>9 | dad_medprob___9 | None of the above
118	add_parent_medprob	Provide any additional information about parent's medical problems	notes
119	num_sib	How many full siblings does your child have?	text
120	num_half_sib	How many half siblings does your child have?	text
121	sib_medprob	Please list your child's full and half siblings, their age and what medical problems they have?<br><br>(1) Recurrent tonsillitis<br>(2) PFAPA<br>(3) OSA diagnosed as a child<br>(4) OSA diagnosed as an adult<br>(5) Tonsillectomy<br>(6) Recurrent otitis media<br>(7) Myringotomy tubes<br>(8) Recurrent aphthous ulcers	notes
122	fhx_possible_pfapa	Does the child have a first degree family member who may have PFAPA?<br>(ie has >5 episodes of fever or tonsillitis in one year)	yesno<br>1 | Yes<br>0 | No
123	fhx_contact_info<br>Show the field ONLY if:<br>[fhx_possible_pfapa] = '1'	If parent or sibling has features suggestive of PFAPA, what is contact information (if any) for who we can contact to get more information	notes

124	mat_gm_medprob	Does your child's MATERNAL GRANDMOTHER have any of the following conditions?	checkbox<br>1 mat_gm_medprob___1 Recurrent tonsillitis<br>2 mat_gm_medprob___2 PFAPA<br>3 mat_gm_medprob___3 OSA diagnosed as a child<br>4 mat_gm_medprob___4 OSA diagnosed as an adult<br>5 mat_gm_medprob___5 Tonsillectomy<br>6 mat_gm_medprob___6 Recurrent otitis media<br>7 mat_gm_medprob___7 Myringotomy tubes<br>8 mat_gm_medprob___8 Recurrent aphthous ulcers<br>9 mat_gm_medprob___9 None of the above
125	mat_gf_medprob	Does your child's MATERNAL GRANDFATHER have any of the following conditions?	checkbox<br>1 mat_gf_medprob___1 Recurrent tonsillitis<br>2 mat_gf_medprob___2 PFAPA<br>3 mat_gf_medprob___3 OSA diagnosed as a child<br>4 mat_gf_medprob___4 OSA diagnosed as an adult<br>5 mat_gf_medprob___5 Tonsillectomy<br>6 mat_gf_medprob___6 Recurrent otitis media<br>7 mat_gf_medprob___7 Myringotomy tubes<br>8 mat_gf_medprob___8 Recurrent aphthous ulcers<br>9 mat_gf_medprob___9 None of the above
126	mat_sib_medprob	Please list your child's mother's full siblings and what medical problems they have?<br><br>(1) Recurrent tonsillitis<br>(2) PFAPA<br>(3) OSA diagnosed as a child<br>(4) OSA diagnosed as an adult<br>(5) Tonsillectomy<br>(6) Recurrent otitis media<br>(7) Myringotomy tubes<br>(8) Recurrent aphthous ulcers	notes
127	pat_gm_medprob	Does your child's PATERNAL GRANDMOTHER have any of the following conditions?	checkbox<br>1 pat_gm_medprob___1 Recurrent tonsillitis<br>2 pat_gm_medprob___2 PFAPA<br>3 pat_gm_medprob___3 OSA diagnosed as a child<br>4 pat_gm_medprob___4 OSA diagnosed as an adult<br>5 pat_gm_medprob___5 Tonsillectomy<br>6 pat_gm_medprob___6 Recurrent otitis media<br>7 pat_gm_medprob___7 Myringotomy tubes<br>8 pat_gm_medprob___8 Recurrent aphthous ulcers<br>9 pat_gm_medprob___9 None of the above

128	pat_gf_medprob	Does your child's PATERNAL GRANDFATHER have any of the following conditions?	checkbox<br>1 pat_gf_medprob___1 Recurrent tonsillitis<br>2 pat_gf_medprob___2 PFAPA<br>3 pat_gf_medprob___3 OSA diagnosed as a child<br>4 pat_gf_medprob___4 OSA diagnosed as an adult<br>5 pat_gf_medprob___5 Tonsillectomy<br>6 pat_gf_medprob___6 Recurrent otitis media<br>7 pat_gf_medprob___7 Myringotomy tubes<br>8 pat_gf_medprob___8 Recurrent aphthous ulcers<br>9 pat_gf_medprob___9 None of the above
129	pat_sib_medprob	Please list your child's father's full siblings and what medical problems they have?<br><br>(1) Recurrent tonsillitis<br>(2) PFAPA<br>(3) OSA diagnosed as a child<br>(4) OSA diagnosed as an adult<br>(5) Tonsillectomy<br>(6) Recurrent otitis media<br>(7) Myringotomy tubes<br>(8) Recurrent aphthous ulcers	notes
130	additional_2deg_medprob	Please provide any additional information about your child's grandparents', aunts', and uncles' medical problems.	notes
131	mother_ethnic_origin	What is your child's mother's ethnic origin (please list all countries)?	notes
132	father_ethnic_origin	What is your child's father's ethnic origin (please list all countries)?	notes
133	mother_occupation	What is the occupation of the child's mother?	radio<br>1 Teacher<br>2 Lawyer<br>3 Doctor<br>4 Nurse<br>5 Pharmacist<br>6 Medical Technician<br>7 Accountant<br>8 Realtor<br>9 Janitor<br>10 Secretary<br>11 Construction Worker<br>12 Other<br>13 Don't know
134	oth_mother_occupation<br>Show the field ONLY if: [mother_occupation] = '12'	Please describe.	text

135	father_occupation	What is the occupation of the child's father?	radio<br>1 Teacher<br>2 Lawyer<br>3 Doctor<br>4 Nurse<br>5 Pharmacist<br>6 Medical Technician<br>7 Accountant<br>8 Realtor<br>9 Janitor<br>10 Secretary<br>11 Construction Worker<br>12 Other<br>13 Don't know
136	oth_father_occupation<br>Show the field ONLY if:<br>[father_occupation] = '12'	Please describe.	text
137	mother_education	What is your child's mother's maximum education level?	radio<br>1 Less than high school<br>2 Graduated from high school<br>3 College degree<br>4 Graduate degree<br>5 Don't know
138	father_education	What is your child's father highest education level?	radio<br>1 Less than high school<br>2 Graduated from high school<br>3 College degree<br>4 Graduate degree<br>5 Don't know
139	osa_18_sleep_disturbance	Section Header: *Quality of Life (OSA-18)*<br>Sleep disturbance	descriptive
140	osa_18_1	During the past 4 weeks, how often has your child had loud snoring?	radio<br>1 None of the time (1)<br>2 Hardly any of the time (2)<br>3 A little of the time (3)<br>4 Some of the time (4)<br>5 A good bit of the time (5)<br>6 Most of the time (6)<br>7 All of the time (7)
141	osa_18_2	During the past 4 weeks, how often has your child had breath-holding spells or pauses in breathing at night?	radio<br>1 None of the time (1)<br>2 Hardly any of the time (2)<br>3 A little of the time (3)<br>4 Some of the time (4)<br>5 A good bit of the time (5)<br>6 Most of the time (6)<br>7 All of the time (7)

142	osa_18_3	During the past 4 weeks, how often has your child had choking or making gasping sounds while asleep?	radio<br>1 None of the time (1)<br>2 Hardly any of the time (2)<br>3 A little of the time (3)<br>4 Some of the time (4)<br>5 A good bit of the time (5)<br>6 Most of the time (6)<br>7 All of the time (7)
143	osa_18_4	During the past 4 weeks, how often has your child had restless sleep or frequent awakening?	radio<br>1 None of the time (1)<br>2 Hardly any of the time (2)<br>3 A little of the time (3)<br>4 Some of the time (4)<br>5 A good bit of the time (5)<br>6 Most of the time (6)<br>7 All of the time (7)
144	osa_18_physical_symptoms	Physical symptoms	descriptive
145	osa_18_5	During the past 4 weeks, how often has your child had mouth breathing because of nasal obstruction?	radio<br>1 None of the time (1)<br>2 Hardly any of the time (2)<br>3 A little of the time (3)<br>4 Some of the time (4)<br>5 A good bit of the time (5)<br>6 Most of the time (6)<br>7 All of the time (7)
146	osa_18_6	During the past 4 weeks, how often has your child had frequent colds or upper respiratory infections?	radio<br>1 None of the time (1)<br>2 Hardly any of the time (2)<br>3 A little of the time (3)<br>4 Some of the time (4)<br>5 A good bit of the time (5)<br>6 Most of the time (6)<br>7 All of the time (7)
147	osa_18_7	During the past 4 weeks, how often has your child had nasal discharge or runny nose?	radio<br>1 None of the time (1)<br>2 Hardly any of the time (2)<br>3 A little of the time (3)<br>4 Some of the time (4)<br>5 A good bit of the time (5)<br>6 Most of the time (6)<br>7 All of the time (7)

148	osa_18_8	During the past 4 weeks, how often has your child had difficulty swallowing?	radio<br>1 None of the time (1)<br>2 Hardly any of the time (2)<br>3 A little of the time (3)<br>4 Some of the time (4)<br>5 A good bit of the time (5)<br>6 Most of the time (6)<br>7 All of the time (7)
149	osa_18_emotional_symptoms	Emotional symptoms	descriptive
150	osa_18_9	During the past 4 weeks, how often has your child had mood swings or temper tantrums?	radio<br>1 None of the time (1)<br>2 Hardly any of the time (2)<br>3 A little of the time (3)<br>4 Some of the time (4)<br>5 A good bit of the time (5)<br>6 Most of the time (6)<br>7 All of the time (7)
151	osa_18_10	During the past 4 weeks, how often has your child had aggressive or hyperactive behavior?	radio<br>1 None of the time (1)<br>2 Hardly any of the time (2)<br>3 A little of the time (3)<br>4 Some of the time (4)<br>5 A good bit of the time (5)<br>6 Most of the time (6)<br>7 All of the time (7)
152	osa_18_11	During the past 4 weeks, how often has your child had discipline problems?	radio<br>1 None of the time (1)<br>2 Hardly any of the time (2)<br>3 A little of the time (3)<br>4 Some of the time (4)<br>5 A good bit of the time (5)<br>6 Most of the time (6)<br>7 All of the time (7)
153	osa_18_daytime_function	Daytime function	descriptive
154	osa_18_12	During the past 4 weeks, how often has your child had excessive daytime sleepiness?	radio<br>1 None of the time (1)<br>2 Hardly any of the time (2)<br>3 A little of the time (3)<br>4 Some of the time (4)<br>5 A good bit of the time (5)<br>6 Most of the time (6)<br>7 All of the time (7)

155	osa_18_13	During the past 4 weeks, how often has your child had poor attention span or concentration?	radio<br>1 None of the time (1)<br>2 Hardly any of the time (2)<br>3 A little of the time (3)<br>4 Some of the time (4)<br>5 A good bit of the time (5)<br>6 Most of the time (6)<br>7 All of the time (7)
156	osa_18_14	During the past 4 weeks, how often has your child had difficulty getting up in the morning?	radio<br>1 None of the time (1)<br>2 Hardly any of the time (2)<br>3 A little of the time (3)<br>4 Some of the time (4)<br>5 A good bit of the time (5)<br>6 Most of the time (6)<br>7 All of the time (7)
157	osa_18_caregiver_concerns	Caregiver concerns	descriptive
158	osa_18_15	During the past 4 weeks, how often have the problems above caused you to worry about your child's general health?	radio<br>1 None of the time (1)<br>2 Hardly any of the time (2)<br>3 A little of the time (3)<br>4 Some of the time (4)<br>5 A good bit of the time (5)<br>6 Most of the time (6)<br>7 All of the time (7)
159	osa_18_16	During the past 4 weeks, how often have the problems above created concern that your child is not getting enough air?	radio<br>1 None of the time (1)<br>2 Hardly any of the time (2)<br>3 A little of the time (3)<br>4 Some of the time (4)<br>5 A good bit of the time (5)<br>6 Most of the time (6)<br>7 All of the time (7)
160	osa_18_17	During the past 4 weeks, how often have the problems above interfered with your ability to perform daily activities?	radio<br>1 None of the time (1)<br>2 Hardly any of the time (2)<br>3 A little of the time (3)<br>4 Some of the time (4)<br>5 A good bit of the time (5)<br>6 Most of the time (6)<br>7 All of the time (7)

161	osa_18_18	During the past 4 weeks, how often have the problems above made you frustrated?	radio<br>1 None of the time (1)<br>2 Hardly any of the time (2)<br>3 A little of the time (3)<br>4 Some of the time (4)<br>5 A good bit of the time (5)<br>6 Most of the time (6)<br>7 All of the time (7)
162	sleep_study	Section Header: *Sleep Study*<br>Did your child have a sleep study?	yesno<br>1 Yes<br>0 No
163	ahi<br>Show the field ONLY if:<br>[sleep_study] = '1'	What was the AHI (apnea-hypopnea index)?	text
164	spo2_nadir<br>Show the field ONLY if:<br>[sleep_study] = '1'	What was the SpO2 nadir?	text
165	email	Section Header: *Contact Information*<br>What is your email address?	text (email)
166	phone	What is the best phone number to reach you?	text
167	ind_prov	Section Header: *Physical Exam and Surgical Information*<br>Per the provider notes, why is this child receiving a tonsillectomy?	checkbox<br>1 ind_prov___1 OSA/sleep disordered breathing<br>2 ind_prov___2 PFAPA<br>3 ind_prov___3 Recurrent tonsillitis<br>4 ind_prov___4 Dysphagia<br>5 ind_prov___5 Dysphonia<br>6 ind_prov___6 Other<br>7 ind_prov___7 Don't know
168	ind_prov_oth<br>Show the field ONLY if:<br>[ind_prov(6)] = '1'	Please describe.	text
169	ulcers	Were aphthous ulcers noted in your child's mouth by physician?	yesno<br>1 Yes<br>0 No
170	cervical_lad	Was cervical lymphadenopathy noted in your child by the physician?	yesno<br>1 Yes<br>0 No
171	tonsil_size	Tonsil size in last ENT physician note (1, 2, 3, 4+).	text
172	height	Child's height (in cm):	text (number, Min: 0)
173	height_per	Height percentile	text
174	weight	Child's weight (in kg):	text (number)
175	weight_per	Weight percentile	text
176	oth_procedures	If your child undergoing any other procedures today in addition to a tonsillectomy?	yesno<br>1 Yes<br>0 No
177	oth_procedures_des<br>Show the field ONLY if:<br>[oth_procedures] = '1'	Which other procedures is the child undergoing today?	checkbox<br>1 oth_procedures_des___1 Adenoidectomy<br>2 oth_procedures_des___2 Myringotomy<br>3 oth_procedures_des___3 Other

178	oth_procedures_exp<br>Show the field ONLY if:<br>[oth_procedures_des(3)] = '1'	Please describe.	notes
179	pfapa_yesno	Does this child meet diagnostic criteria for PFAPA?	radio<br>1 Definite<br>2 Probable<br>3 Possible<br>4 No
180	path_num	What is the pathology number of the tonsil specimen?	text
181	datet	Date of Tonsillectomy	text (date_mdy)
182	date	Date of Survey	text (date_mdy)
183	initial_survey_complete	Section Header: *Form Status*<br>Complete?	dropdown<br>0 Incomplete<br>1 Unverified<br>2 Complete
Instrument: **3 Month Follow Up Survey** (month_follow_up_survey)			Collapse
184	surg_comp3	Section Header: *Surgical Course*<br>Since tonsillectomy, has your child been taken to the emergency department or admitted to the hospital due to a complication of his/her tonsillectomy?	yesno<br>1 Yes<br>0 No
185	surg_comp_exp3<br>Show the field ONLY if:<br>[surg_comp3]='1'	Why was your child taken to the emergency department or admitted to the hospital after tonsillectomy?	checkbox<br>1 surg_comp_exp3___1 Bleeding<br>2 surg_comp_exp3___2 Dehydration<br>3 surg_comp_exp3___3 Pain Control<br>4 surg_comp_exp3___4 Other
186	surg_comp_exp_oth3<br>Show the field ONLY if:<br>[surg_comp_exp3(4)]='1'	Please describe "other"	notes
187	new_mh3	Section Header: *Medical History*<br>Has your child been diagnosed with any new medical conditions since tonsillectomy?	yesno<br>1 Yes<br>0 No
188	info_mh3<br>Show the field ONLY if:<br>[new_mh3] = '1'	Please provide more detail about your child's new medical conditions.	notes
189	hosp3	Has your child been hospitalized since tonsillectomy for any reason other than a complication of his/her tonsillectomy?	yesno<br>1 Yes<br>0 No
190	info_hosp3<br>Show the field ONLY if:<br>[hosp3] = '1'	Please provide more detail about your child's hospitalization(s).	notes
191	new_sh3	Has your child undergone any surgeries or procedures since tonsillectomy?	yesno<br>1 Yes<br>0 No
192	info_sh3<br>Show the field ONLY if:<br>[new_sh3] = '1'	Please provide more detail about your child's surgeries and/or procedures.	notes
193	ton_num3	Section Header: *Recurrent tonsillitis*<br>Since tonsillectomy, how many throat or tonsil infections has your child had?	text (integer)

194	ton_wfev3<br>Show the field ONLY if:<br>[ton_num3] >= 1	For how many of these episodes did your child have fever of 101 degrees F or higher?	text
195	ton_wstrep_pos3<br>Show the field ONLY if:<br>[ton_num3] >= 1	For how many of these episodes did your child have positive testing for strep?	text
196	rec_ton3<br>Show the field ONLY if:<br>[ton_num3] >= 1	When was the most recent episode? Please provide the date if known, otherwise estimate.	text
197	ton_as_sym3<br>Show the field ONLY if:<br>[ton_num3] >= 1	What symptoms did your child have when he/she had a throat or tonsil infection? Check all that apply:	checkbox<br>1 ton_as_sym3___1 Sore throat<br>2 ton_as_sym3___2 Swollen lymph nodes in neck<br>3 ton_as_sym3___3 Mouth ulcers<br>4 ton_as_sym3___4 Red tonsils<br>5 ton_as_sym3___5 Pus/white spots on tonsils<br>6 ton_as_sym3___6 Headache<br>7 ton_as_sym3___7 Abdominal pain<br>8 ton_as_sym3___8 Muscle aches<br>9 ton_as_sym3___9 Other
198	oth_ton_as_sym3<br>Show the field ONLY if:<br>[ton_as_sym3(9)] = '1'	Please describe "other"	text
199	add_ton3<br>Show the field ONLY if:<br>[ton_num3] >= 1	Please provide any additional information on tonsillitis episodes.	notes
200	psq1_3	Section Header: *Pediatric Sleep Questionnaire*<br>When sleeping, does your child snore more than half the time?	radio<br>1 Yes<br>0 No<br>2 Don't know
201	psq2_3	When sleeping, does your child always snore?	radio<br>1 Yes<br>0 No<br>2 Don't know
202	psq3_3	When sleeping, does your child snore loudly?	radio<br>1 Yes<br>0 No<br>2 Don't know
203	psq4_3	When sleeping, does your child have "heavy" or loud breathing?	radio<br>1 Yes<br>0 No<br>2 Don't know
204	psq5_3	When sleeping, does your child have trouble breathing, or struggle to breathe?	radio<br>1 Yes<br>0 No<br>2 Don't know
205	psq6_3	Have you ever seen your child stop breathing during the night?	radio<br>1 Yes<br>0 No<br>2 Don't know

206	psq7_3	Does your child tend to breathe through the mouth during the day?	radio<br>1 Yes<br>0 No<br>2 Don't know
207	psq8_3	Does your child have a dry mouth on waking up in the morning?	radio<br>1 Yes<br>0 No<br>2 Don't know
208	psq9_3	Does your child occasionally wet the bed?	radio<br>1 Yes<br>0 No<br>2 Don't know
209	psq10_3	Does your child wake up feeling unrefreshed in the morning?	radio<br>1 Yes<br>0 No<br>2 Don't know
210	psq11_3	Does your child have a problem with sleepiness during the day?	radio<br>1 Yes<br>0 No<br>2 Don't know
211	psq12_3	Has a teacher or other supervisor commented that your child appears sleepy during the day?	radio<br>1 Yes<br>0 No<br>2 Don't know
212	psq13_3	Is it hard to wake your child up in the morning?	radio<br>1 Yes<br>0 No<br>2 Don't know
213	psq14_3	Does your child wake up with headaches in the morning?	radio<br>1 Yes<br>0 No<br>2 Don't know
214	psq15_3	Did your child stop growing at a normal rate at any time since birth?	radio<br>1 Yes<br>0 No<br>2 Don't know
215	psq16_3	Is your child overweight?	radio<br>1 Yes<br>0 No<br>2 Don't know
216	psq17_3	Does your child often not seem to listen when spoken to directly?	radio<br>1 Yes<br>0 No<br>2 Don't know
217	psq18_3	Does your child often have difficulty organizing tasks and activities?	radio<br>1 Yes<br>0 No<br>2 Don't know

218	psq19_3	Is your child often easily distracted by extraneous stimuli?	radio<br>1 Yes<br>0 No<br>2 Don't know
219	psq20_3	Does your child fidget with his/her hands or feet or squirms in his/her seat?	radio<br>1 Yes<br>0 No<br>2 Don't know
220	psq21_3	Is your child 'on the go' or often act as if 'driven by a motor'?	radio<br>1 Yes<br>0 No<br>2 Don't know
221	psq22_3	Does your child often interrupt or intrude on others (e.g. butts into conversations or games)?	radio<br>1 Yes<br>0 No<br>2 Don't know
222	canker_sores_or_ulcers3	Section Header: *Other Symptoms*<br>Has your child had canker sores or ulcers in his/her mouth since tonsillectomy?	radio<br>1 Yes<br>0 No<br>2 Don't know
223	ear_infxn3	How many ear infections has your child had since tonsillectomy?	text
224	dysphagia3	Has your child had difficulty swallowing since the tonsillectomy?	yesno<br>1 Yes<br>0 No
225	dysphagia_as_sym3<br>Show the field ONLY if:<br>[dysphagia3] = '1'	Is your child's difficulty swallowing related to any of the following symptoms? Please check all that apply.	checkbox<br>1 dysphagia_as_sym3___1 Fever<br>2 dysphagia_as_sym3___2 Tonsillitis<br>3 dysphagia_as_sym3___3 Canker sores or mouth ulcers<br>4 dysphagia_as_sym3___4 Ear infection<br>5 dysphagia_as_sym3___5 Large tonsils<br>6 dysphagia_as_sym3___6 Don't know<br>7 dysphagia_as_sym3___7 Other
226	dysphagia_as_sym_oth3<br>Show the field ONLY if:<br>[dysphagia_as_sym3(7)]='1'	Describe other.	text
227	dysphonia3	Has your child had trouble speaking since the tonsillectomy?	yesno<br>1 Yes<br>0 No
228	dysphonia_as_sym3<br>Show the field ONLY if:<br>[dysphonia3] = '1'	Is your child's difficulty speaking related to any of the following symptoms? Please check all that apply.	checkbox<br>1 dysphonia_as_sym3___1 Fever<br>2 dysphonia_as_sym3___2 Tonsillitis<br>3 dysphonia_as_sym3___3 Canker sores or mouth ulcers<br>4 dysphonia_as_sym3___4 Ear infection<br>5 dysphonia_as_sym3___5 Large tonsils<br>6 dysphonia_as_sym3___6 Don't know<br>7 dysphonia_as_sym3___7 Other

229	dysphonia_as_sym_oth3<br>Show the field ONLY if: [dysphonia_as_sym3(7)]='1'	Describe other.	text
230	speech_tx3	Has your child received speech therapy since tonsillectomy?	yesno<br>1 Yes<br>0 No
231	sdays_miss3	How many days of school has your child missed since tonsillectomy?	text (integer)
232	sdays_miss_ton3	How many of these missed school days were due to tonsil problems?	text
233	num_abx3<br>Show the field ONLY if: [ton_num3] >= 1	How many times has your child gotten a course of antibiotics since tonsillectomy?	text
234	osa_18_sleep_disturbance3	Section Header: *Quality of Life (OSA-18)*<br>Sleep disturbance	descriptive
235	osa_18_1_3	During the past 4 weeks, how often has your child had loud snoring?	radio<br>1 None of the time (1)<br>2 Hardly any of the time (2)<br>3 A little of the time (3)<br>4 Some of the time (4)<br>5 A good bit of the time (5)<br>6 Most of the time (6)<br>7 All of the time (7)
236	osa_18_2_3	During the past 4 weeks, how often has your child had breath-holding spells or pauses in breathing at night?	radio<br>1 None of the time (1)<br>2 Hardly any of the time (2)<br>3 A little of the time (3)<br>4 Some of the time (4)<br>5 A good bit of the time (5)<br>6 Most of the time (6)<br>7 All of the time (7)
237	osa_18_3_3	During the past 4 weeks, how often has your child had choking or making gasping sounds while asleep?	radio<br>1 None of the time (1)<br>2 Hardly any of the time (2)<br>3 A little of the time (3)<br>4 Some of the time (4)<br>5 A good bit of the time (5)<br>6 Most of the time (6)<br>7 All of the time (7)
238	osa_18_4_3	During the past 4 weeks, how often has your child had restless sleep or frequent awakening?	radio<br>1 None of the time (1)<br>2 Hardly any of the time (2)<br>3 A little of the time (3)<br>4 Some of the time (4)<br>5 A good bit of the time (5)<br>6 Most of the time (6)<br>7 All of the time (7)
239	osa_18_physical_symptoms_3	Physical symptoms	descriptive

240	osa_18_5_3	During the past 4 weeks, how often has your child had mouth breathing because of nasal obstruction?	radio<br>1 None of the time (1)<br>2 Hardly any of the time (2)<br>3 A little of the time (3)<br>4 Some of the time (4)<br>5 A good bit of the time (5)<br>6 Most of the time (6)<br>7 All of the time (7)
241	osa_18_6_3	During the past 4 weeks, how often has your child had frequent colds or upper respiratory infections?	radio<br>1 None of the time (1)<br>2 Hardly any of the time (2)<br>3 A little of the time (3)<br>4 Some of the time (4)<br>5 A good bit of the time (5)<br>6 Most of the time (6)<br>7 All of the time (7)
242	osa_18_7_3	During the past 4 weeks, how often has your child had nasal discharge or runny nose?	radio<br>1 None of the time (1)<br>2 Hardly any of the time (2)<br>3 A little of the time (3)<br>4 Some of the time (4)<br>5 A good bit of the time (5)<br>6 Most of the time (6)<br>7 All of the time (7)
243	osa_18_8_3	During the past 4 weeks, how often has your child had difficulty swallowing?	radio<br>1 None of the time (1)<br>2 Hardly any of the time (2)<br>3 A little of the time (3)<br>4 Some of the time (4)<br>5 A good bit of the time (5)<br>6 Most of the time (6)<br>7 All of the time (7)
244	osa_18_emotional_symptoms_3	Emotional symptoms	descriptive
245	osa_18_9_3	During the past 4 weeks, how often has your child had mood swings or temper tantrums?	radio<br>1 None of the time (1)<br>2 Hardly any of the time (2)<br>3 A little of the time (3)<br>4 Some of the time (4)<br>5 A good bit of the time (5)<br>6 Most of the time (6)<br>7 All of the time (7)

246	osa_18_10_3	During the past 4 weeks, how often has your child had aggressive or hyperactive behavior?	radio<br>1 None of the time (1)<br>2 Hardly any of the time (2)<br>3 A little of the time (3)<br>4 Some of the time (4)<br>5 A good bit of the time (5)<br>6 Most of the time (6)<br>7 All of the time (7)
247	osa_18_11_3	During the past 4 weeks, how often has your child had discipline problems?	radio<br>1 None of the time (1)<br>2 Hardly any of the time (2)<br>3 A little of the time (3)<br>4 Some of the time (4)<br>5 A good bit of the time (5)<br>6 Most of the time (6)<br>7 All of the time (7)
248	osa_18_daytime_function_3	Daytime function	descriptive
249	osa_18_12_3	During the past 4 weeks, how often has your child had excessive daytime sleepiness?	radio<br>1 None of the time (1)<br>2 Hardly any of the time (2)<br>3 A little of the time (3)<br>4 Some of the time (4)<br>5 A good bit of the time (5)<br>6 Most of the time (6)<br>7 All of the time (7)
250	osa_18_13_3	During the past 4 weeks, how often has your child had poor attention span or concentration?	radio<br>1 None of the time (1)<br>2 Hardly any of the time (2)<br>3 A little of the time (3)<br>4 Some of the time (4)<br>5 A good bit of the time (5)<br>6 Most of the time (6)<br>7 All of the time (7)
251	osa_18_14_3	During the past 4 weeks, how often has your child had difficulty getting up in the morning?	radio<br>1 None of the time (1)<br>2 Hardly any of the time (2)<br>3 A little of the time (3)<br>4 Some of the time (4)<br>5 A good bit of the time (5)<br>6 Most of the time (6)<br>7 All of the time (7)
252	osa_18_caregiver_concerns_3	Caregiver concerns	descriptive

253	osa_18_15_3	During the past 4 weeks, how often have the problems above caused you to worry about your child's general health?	radio<br>1 None of the time (1)<br>2 Hardly any of the time (2)<br>3 A little of the time (3)<br>4 Some of the time (4)<br>5 A good bit of the time (5)<br>6 Most of the time (6)<br>7 All of the time (7)
254	osa_18_16_3	During the past 4 weeks, how often have the problems above created concern that your child is not getting enough air?	radio<br>1 None of the time (1)<br>2 Hardly any of the time (2)<br>3 A little of the time (3)<br>4 Some of the time (4)<br>5 A good bit of the time (5)<br>6 Most of the time (6)<br>7 All of the time (7)
255	osa_18_17_3	During the past 4 weeks, how often have the problems above interfered with your ability to perform daily activities?	radio<br>1 None of the time (1)<br>2 Hardly any of the time (2)<br>3 A little of the time (3)<br>4 Some of the time (4)<br>5 A good bit of the time (5)<br>6 Most of the time (6)<br>7 All of the time (7)
256	osa_18_18_3	During the past 4 weeks, how often have the problems above made you frustrated?	radio<br>1 None of the time (1)<br>2 Hardly any of the time (2)<br>3 A little of the time (3)<br>4 Some of the time (4)<br>5 A good bit of the time (5)<br>6 Most of the time (6)<br>7 All of the time (7)
257	sleep_study3	Section Header: *Sleep Study*<br>Has your child had a sleep study since tonsillectomy?	yesno<br>1 Yes<br>0 No
258	ahi3<br>Show the field ONLY if: [sleep_study3] = '1'	What was the AHI (apnea-hypopnea index)? If you don't know, please leave blank.	text
259	rdi3<br>Show the field ONLY if: [sleep_study3] = '1'	What was the RDI (respiratory disturbance index)? If you don't know, please leave blank.	text
260	spo2_nadir3<br>Show the field ONLY if: [sleep_study3] = '1'	What was the SpO2 nadir? If you don't know, please leave blank.	text
261	height3	Section Header: *Height and Weight*<br>What is your child's most recent height in inches? If you don't know, please leave blank.	text (number, Min: 0)
262	weight3	What is your child's most recent weight in pounds? If you don't know, please leave blank.	text (number)

263	date3	Section Header: *Date*<br>Date of Survey	text (date_mdy)
264	month_follow_up_survey_complete	Section Header: *Form Status*<br>Complete?	dropdown<br>0 Incomplete<br>1 Unverified<br>2 Complete

Instrument: **6 Month Follow Up Survey** (month_follow_up_survey_ec1e)

Collapse

265	new_mh6	Section Header: *Medical History*<br>Has your child been diagnosed with any new medical conditions in the last 3 months?	yesno<br>1 Yes<br>0 No
266	info_mh6<br>Show the field ONLY if:<br>[new_mh6] = '1'	Please provide more detail about your child's new medical conditions.	notes
267	hosp6	Has your child been hospitalized in the last 3 months?	yesno<br>1 Yes<br>0 No
268	info_hosp6<br>Show the field ONLY if:<br>[hosp6] = '1'	Please provide more detail about your child's hospitalization(s).	notes
269	new_sh6	Has your child undergone any surgeries or procedures in the in the last 3 months?	yesno<br>1 Yes<br>0 No
270	info_sh6<br>Show the field ONLY if:<br>[new_sh6] = '1'	Please provide more detail about your child's surgeries and/or procedures.	notes
271	ton_num6	Section Header: *Recurrent tonsillitis*<br>In the last 3 months, how many throat or tonsil infections has your child had?	text (integer)
272	ton_wfev6<br>Show the field ONLY if:<br>[ton_num6] >= 1	For how many of these episodes did your child have fever of 101 degrees F or higher?	text
273	ton_wstrep_pos6<br>Show the field ONLY if:<br>[ton_num6] >= 1	For how many of these episodes did your child have positive testing for strep?	text
274	rec_ton6<br>Show the field ONLY if:<br>[ton_num6] >= 1	When was the most recent episode? Please provide the date if known, otherwise estimate.	text
275	ton_as_sym6<br>Show the field ONLY if:<br>[ton_num6] >= 1	What symptoms did your child have when he/she had a throat or tonsil infection? Check all that apply:	checkbox<br>1 ton_as_sym6___1 Sore throat<br>2 ton_as_sym6___2 Swollen lymph nodes in neck<br>3 ton_as_sym6___3 Mouth ulcers<br>4 ton_as_sym6___4 Red tonsils<br>5 ton_as_sym6___5 Pus/white spots on tonsils<br>6 ton_as_sym6___6 Headache<br>7 ton_as_sym6___7 Abdominal pain<br>8 ton_as_sym6___8 Muscle aches<br>9 ton_as_sym6___9 Other
276	oth_ton_as_sym6<br>Show the field ONLY if:<br>[ton_as_sym6(9)] = '1'	Please describe "other"	text

277	add_ton6<br>Show the field ONLY if:<br>[ton_num6] >= 1	Please provide any additional information on tonsillitis episodes.	notes
278	psq1_6	Section Header: *Pediatric Sleep Questionnaire*<br>When sleeping, does your child snore more than half the time?	radio<br>1 Yes<br>0 No<br>2 Don't know
279	psq2_6	When sleeping, does your child always snore?	radio<br>1 Yes<br>0 No<br>2 Don't know
280	psq3_6	When sleeping, does your child snore loudly?	radio<br>1 Yes<br>0 No<br>2 Don't know
281	psq4_6	When sleeping, does your child have "heavy" or loud breathing?	radio<br>1 Yes<br>0 No<br>2 Don't know
282	psq5_6	When sleeping, does your child have trouble breathing, or struggle to breathe?	radio<br>1 Yes<br>0 No<br>2 Don't know
283	psq6_6	Have you ever seen your child stop breathing during the night?	radio<br>1 Yes<br>0 No<br>2 Don't know
284	psq7_6	Does your child tend to breathe through the mouth during the day?	radio<br>1 Yes<br>0 No<br>2 Don't know
285	psq8_6	Does your child have a dry mouth on waking up in the morning?	radio<br>1 Yes<br>0 No<br>2 Don't know
286	psq9_6	Does your child occasionally wet the bed?	radio<br>1 Yes<br>0 No<br>2 Don't know or child too young
287	psq10_6	Does your child wake up feeling unrefreshed in the morning?	radio<br>1 Yes<br>0 No<br>2 Don't know
288	psq11_6	Does your child have a problem with sleepiness during the day?	radio<br>1 Yes<br>0 No<br>2 Don't know

289	psq12_6	Has a teacher or other supervisor commented that your child appears sleepy during the day?	radio<br>1 Yes<br>0 No<br>2 Don't know
290	psq13_6	Is it hard to wake your child up in the morning?	radio<br>1 Yes<br>0 No<br>2 Don't know
291	psq14_6	Does your child wake up with headaches in the morning?	radio<br>1 Yes<br>0 No<br>2 Don't know
292	psq15_6	Did your child stop growing at a normal rate at any time since birth?	radio<br>1 Yes<br>0 No<br>2 Don't know
293	psq16_6	Is your child overweight?	radio<br>1 Yes<br>0 No<br>2 Don't know
294	psq17_6	Does your child often not seem to listen when spoken to directly?	radio<br>1 Yes<br>0 No<br>2 Don't know
295	psq18_6	Does your child often have difficulty organizing tasks and activities?	radio<br>1 Yes<br>0 No<br>2 Don't know
296	psq19_6	Is your child often easily distracted by extraneous stimuli?	radio<br>1 Yes<br>0 No<br>2 Don't know
297	psq20_6	Does your child fidget with his/her hands or feet or squirms in his/her seat?	radio<br>1 Yes<br>0 No<br>2 Don't know
298	psq21_6	Is your child 'on the go' or often act as if 'driven by a motor'?	radio<br>1 Yes<br>0 No<br>2 Don't know
299	psq22_6	Does your child often interrupt or intrude on others (e.g. butts into conversations or games)?	radio<br>1 Yes<br>0 No<br>2 Don't know
300	canker_sores_or_ulcers6	Section Header: *Other Symptoms*<br>Has your child had canker sores or ulcers in his/her mouth in the last 3 months?	radio<br>1 Yes<br>0 No<br>2 Don't know

301	ear_infxn6	How many ear infections has your child had since the last questionnaire (last 3 months)?	text
302	dysphagia6	Has your child had difficulty swallowing since the last questionnaire (last 3 months)?	yesno<br>1 Yes<br>0 No
303	dysphagia_as_sym6<br>Show the field ONLY if:<br>[dysphagia6] = '1'	Is your child's difficulty swallowing related to any of the following symptoms? Please check all that apply.	checkbox<br>1 dysphagia_as_sym6___1 Fever<br>2 dysphagia_as_sym6___2 Tonsillitis<br>3 dysphagia_as_sym6___3 Canker sores or mouth ulcers<br>4 dysphagia_as_sym6___4 Ear infection<br>5 dysphagia_as_sym6___5 Large tonsils<br>6 dysphagia_as_sym6___6 Don't know<br>7 dysphagia_as_sym6___7 Other
304	dysphagia_as_sym_oth6<br>Show the field ONLY if:<br>[dysphagia_as_sym6(7)]='1'	Describe other.	text
305	dysphonia6	Has your child had difficulty speaking since the last questionnaire (3 months)?	yesno<br>1 Yes<br>0 No
306	dysphonia_as_sym6<br>Show the field ONLY if:<br>[dysphonia6] = '1'	Is your child's difficulty speaking related to any of the following symptoms? Please check all that apply.	checkbox<br>1 dysphonia_as_sym6___1 Fever<br>2 dysphonia_as_sym6___2 Tonsillitis<br>3 dysphonia_as_sym6___3 Canker sores or mouth ulcers<br>4 dysphonia_as_sym6___4 Ear infection<br>5 dysphonia_as_sym6___5 Large tonsils<br>6 dysphonia_as_sym6___6 Don't know<br>7 dysphonia_as_sym6___7 Other
307	dysphonia_as_sym_oth6<br>Show the field ONLY if:<br>[dysphonia_as_sym6(7)]='1'	Describe other.	text
308	speech_tx6	Has your child received speech therapy in the last 3 months?	yesno<br>1 Yes<br>0 No
309	sdays_miss6	How many days of school has your child missed since the last questionnaire (3 months)?	text (integer)
310	sdays_miss_ton6	How many of these missed school days were due to tonsil problems?	text
311	num_abx6<br>Show the field ONLY if:<br>[ton_num6] >= 1	How many times has your child gotten a course of antibiotics in the last 3 months?	text
312	osa_18_sleep_disturbance6	Section Header: *Quality of Life (OSA-18)*<br>Sleep disturbance	descriptive

313	osa_18_1_6	During the past 4 weeks, how often has your child had loud snoring?	radio<br>1 None of the time (1)<br>2 Hardly any of the time (2)<br>3 A little of the time (3)<br>4 Some of the time (4)<br>5 A good bit of the time (5)<br>6 Most of the time (6)<br>7 All of the time (7)
314	osa_18_2_6	During the past 4 weeks, how often has your child had breath-holding spells or pauses in breathing at night?	radio<br>1 None of the time (1)<br>2 Hardly any of the time (2)<br>3 A little of the time (3)<br>4 Some of the time (4)<br>5 A good bit of the time (5)<br>6 Most of the time (6)<br>7 All of the time (7)
315	osa_18_3_6	During the past 4 weeks, how often has your child had choking or making gasping sounds while asleep?	radio<br>1 None of the time (1)<br>2 Hardly any of the time (2)<br>3 A little of the time (3)<br>4 Some of the time (4)<br>5 A good bit of the time (5)<br>6 Most of the time (6)<br>7 All of the time (7)
316	osa_18_4_6	During the past 4 weeks, how often has your child had restless sleep or frequent awakening?	radio<br>1 None of the time (1)<br>2 Hardly any of the time (2)<br>3 A little of the time (3)<br>4 Some of the time (4)<br>5 A good bit of the time (5)<br>6 Most of the time (6)<br>7 All of the time (7)
317	osa_18_physical_symptoms_6	Physical symptoms	descriptive
318	osa_18_5_6	During the past 4 weeks, how often has your child had mouth breathing because of nasal obstruction?	radio<br>1 None of the time (1)<br>2 Hardly any of the time (2)<br>3 A little of the time (3)<br>4 Some of the time (4)<br>5 A good bit of the time (5)<br>6 Most of the time (6)<br>7 All of the time (7)

319	osa_18_6_6	During the past 4 weeks, how often has your child had frequent colds or upper respiratory infections?	radio<br>1 None of the time (1)<br>2 Hardly any of the time (2)<br>3 A little of the time (3)<br>4 Some of the time (4)<br>5 A good bit of the time (5)<br>6 Most of the time (6)<br>7 All of the time (7)
320	osa_18_7_6	During the past 4 weeks, how often has your child had nasal discharge or runny nose?	radio<br>1 None of the time (1)<br>2 Hardly any of the time (2)<br>3 A little of the time (3)<br>4 Some of the time (4)<br>5 A good bit of the time (5)<br>6 Most of the time (6)<br>7 All of the time (7)
321	osa_18_8_6	During the past 4 weeks, how often has your child had difficulty swallowing?	radio<br>1 None of the time (1)<br>2 Hardly any of the time (2)<br>3 A little of the time (3)<br>4 Some of the time (4)<br>5 A good bit of the time (5)<br>6 Most of the time (6)<br>7 All of the time (7)
322	osa_18_emotional_symptoms_6	Emotional symptoms	descriptive
323	osa_18_9_6	During the past 4 weeks, how often has your child had mood swings or temper tantrums?	radio<br>1 None of the time (1)<br>2 Hardly any of the time (2)<br>3 A little of the time (3)<br>4 Some of the time (4)<br>5 A good bit of the time (5)<br>6 Most of the time (6)<br>7 All of the time (7)
324	osa_18_10_6	During the past 4 weeks, how often has your child had aggressive or hyperactive behavior?	radio<br>1 None of the time (1)<br>2 Hardly any of the time (2)<br>3 A little of the time (3)<br>4 Some of the time (4)<br>5 A good bit of the time (5)<br>6 Most of the time (6)<br>7 All of the time (7)

325	osa_18_11_6	During the past 4 weeks, how often has your child had discipline problems?	radio<br>1 None of the time (1)<br>2 Hardly any of the time (2)<br>3 A little of the time (3)<br>4 Some of the time (4)<br>5 A good bit of the time (5)<br>6 Most of the time (6)<br>7 All of the time (7)
326	osa_18_daytime_function_6	Daytime function	descriptive
327	osa_18_12_6	During the past 4 weeks, how often has your child had excessive daytime sleepiness?	radio<br>1 None of the time (1)<br>2 Hardly any of the time (2)<br>3 A little of the time (3)<br>4 Some of the time (4)<br>5 A good bit of the time (5)<br>6 Most of the time (6)<br>7 All of the time (7)
328	osa_18_13_6	During the past 4 weeks, how often has your child had poor attention span or concentration?	radio<br>1 None of the time (1)<br>2 Hardly any of the time (2)<br>3 A little of the time (3)<br>4 Some of the time (4)<br>5 A good bit of the time (5)<br>6 Most of the time (6)<br>7 All of the time (7)
329	osa_18_14_6	During the past 4 weeks, how often has your child had difficulty getting up in the morning?	radio<br>1 None of the time (1)<br>2 Hardly any of the time (2)<br>3 A little of the time (3)<br>4 Some of the time (4)<br>5 A good bit of the time (5)<br>6 Most of the time (6)<br>7 All of the time (7)
330	osa_18_caregiver_concerns_6	Caregiver concerns	descriptive
331	osa_18_15_6	During the past 4 weeks, how often have the problems above caused you to worry about your child's general health?	radio<br>1 None of the time (1)<br>2 Hardly any of the time (2)<br>3 A little of the time (3)<br>4 Some of the time (4)<br>5 A good bit of the time (5)<br>6 Most of the time (6)<br>7 All of the time (7)

332	osa_18_16_6	During the past 4 weeks, how often have the problems above created concern that your child is not getting enough air?	radio<br>1 None of the time (1)<br>2 Hardly any of the time (2)<br>3 A little of the time (3)<br>4 Some of the time (4)<br>5 A good bit of the time (5)<br>6 Most of the time (6)<br>7 All of the time (7)
333	osa_18_17_6	During the past 4 weeks, how often have the problems above interfered with your ability to perform daily activities?	radio<br>1 None of the time (1)<br>2 Hardly any of the time (2)<br>3 A little of the time (3)<br>4 Some of the time (4)<br>5 A good bit of the time (5)<br>6 Most of the time (6)<br>7 All of the time (7)
334	osa_18_18_6	During the past 4 weeks, how often have the problems above made you frustrated?	radio<br>1 None of the time (1)<br>2 Hardly any of the time (2)<br>3 A little of the time (3)<br>4 Some of the time (4)<br>5 A good bit of the time (5)<br>6 Most of the time (6)<br>7 All of the time (7)
335	sleep_study6	Section Header: *Sleep Study*<br>Has your child had a sleep study in the last 3 months?	yesno<br>1 Yes<br>0 No
336	ahi6<br>Show the field ONLY if:<br>[sleep_study6] = '1'	What was the AHI (apnea-hypopnea index)? If you do not know, please leave blank.	text
337	rdi6<br>Show the field ONLY if:<br>[sleep_study6] = '1'	What was the RDI (respiratory disturbance index)? If you do not know, please leave blank.	text
338	spo2_nadir6<br>Show the field ONLY if:<br>[sleep_study6] = '1'	What was the SpO2 nadir? If you do not know, please leave blank.	text
339	height6	Section Header: *Height and Weight*<br>What is your child's current height (in inches). If you do not know, please leave blank.	text
340	weight6	What is your child's current weight (in lbs)? If you do not know, please leave blank.	text
341	date6	Section Header: *Date*<br>Date of Survey	text (date_mdy)
342	month_follow_up_survey_ec1e_complete	Section Header: *Form Status*<br>Complete?	dropdown<br>0 Incomplete<br>1 Unverified<br>2 Complete

Instrument: **12 Month Follow Up Survey** (month_follow_up_survey_88ee) Collapse

343	new_mh12	Section Header: *Medical History*<br>Has your child been diagnosed with any new medical conditions in the last 12 months?	yesno<br>1 Yes<br>0 No
344	info_mh12<br>Show the field ONLY if:<br>[new_mh12] = '1'	Please provide more detail about your child's new medical conditions.	notes
345	hosp12	Has your child been hospitalized in the last 12 months?	yesno<br>1 Yes<br>0 No
346	info_hosp12<br>Show the field ONLY if:<br>[hosp12] = '1'	Please provide more detail about your child's hospitalization(s).	notes
347	new_sh12	Has your child undergone any surgeries or procedures in the in the last 12 months?	yesno<br>1 Yes<br>0 No
348	info_sh12<br>Show the field ONLY if:<br>[new_sh12] = '1'	Please provide more detail about your child's surgeries and/or procedures.	notes
349	ton_num12	Section Header: *Recurrent tonsillitis*<br>In the last 12 months, how many throat or tonsil infections has your child had?	text (integer)
350	ton_wfev12<br>Show the field ONLY if:<br>[ton_num12] >= 1	For how many of these episodes did your child have fever of 101 degrees F or higher?	text
351	ton_wstrep_pos12<br>Show the field ONLY if:<br>[ton_num12] >= 1	For how many of these episodes did your child have positive testing for strep?	text
352	rec_ton12<br>Show the field ONLY if:<br>[ton_num12] >= 1	When was the most recent episode? Please provide the date if known, otherwise estimate.	text
353	ton_as_sym12<br>Show the field ONLY if:<br>[ton_num12] >= 1	What symptoms did your child have when he/she had a throat or tonsil infection? Check all that apply:	checkbox<br>1 ton_as_sym12___1 Sore throat<br>2 ton_as_sym12___2 Swollen lymph nodes in neck<br>3 ton_as_sym12___3 Mouth ulcers<br>4 ton_as_sym12___4 Red tonsils<br>5 ton_as_sym12___5 Pus/white spots on tonsils<br>6 ton_as_sym12___6 Headache<br>7 ton_as_sym12___7 Abdominal pain<br>8 ton_as_sym12___8 Muscle aches<br>9 ton_as_sym12___9 Other
354	oth_ton_as_sym12<br>Show the field ONLY if:<br>[ton_as_sym12(9)] = '1'	Please describe "other"	text
355	ton_sym_same_12<br>Show the field ONLY if:<br>[ton_num12] >= 1	Are your child's throat and tonsil infections after tonsillectomy different from those he/she had before tonsillectomy in terms of symptoms?	radio<br>1 Yes, episodes before and after tonsillectomy are different<br>2 No, episodes before and after tonsillectomy are the same<br>3 My child did not have any throat or tonsil infections before tonsillectomy<br>4 Don't know

356	ton_sym_diff_12<br>Show the field ONLY if:<br>[ton_sym_same_12] = '1'	Please describe how the episodes are different than those prior to tonsillectomy.	notes
357	add_ton12<br>Show the field ONLY if:<br>[ton_num12] >= 1	Please provide any additional information on tonsillitis episodes.	notes
358	psq1_12	Section Header: *Pediatric Sleep Questionnaire*<br>When sleeping, does your child snore more than half the time?	radio<br>1 Yes<br>0 No<br>2 Don't know
359	psq2_12	When sleeping, does your child always snore?	radio<br>1 Yes<br>0 No<br>2 Don't know
360	psq3_12	When sleeping, does your child snore loudly?	radio<br>1 Yes<br>0 No<br>2 Don't know
361	psq4_12	When sleeping, does your child have "heavy" or loud breathing?	radio<br>1 Yes<br>0 No<br>2 Don't know
362	psq5_12	When sleeping, does your child have trouble breathing, or struggle to breathe?	radio<br>1 Yes<br>0 No<br>2 Don't know
363	psq6_12	Have you ever seen your child stop breathing during the night?	radio<br>1 Yes<br>0 No<br>2 Don't know
364	psq7_12	Does your child tend to breathe through the mouth during the day?	radio<br>1 Yes<br>0 No<br>2 Don't know
365	psq8_12	Does your child have a dry mouth on waking up in the morning?	radio<br>1 Yes<br>0 No<br>2 Don't know
366	psq9_12	Does your child occasionally wet the bed?	radio<br>1 Yes<br>0 No<br>2 Don't know
367	psq10_12	Does your child wake up feeling unrefreshed in the morning?	radio<br>1 Yes<br>0 No<br>2 Don't know

368	psq11_12	Does your child have a problem with sleepiness during the day?	radio<br>1 Yes<br>0 No<br>2 Don't know
369	psq12_12	Has a teacher or other supervisor commented that your child appears sleepy during the day?	radio<br>1 Yes<br>0 No<br>2 Don't know
370	psq13_12	Is it hard to wake your child up in the morning?	radio<br>1 Yes<br>0 No<br>2 Don't know
371	psq14_12	Does your child wake up with headaches in the morning?	radio<br>1 Yes<br>0 No<br>2 Don't know
372	psq15_12	Did your child stop growing at a normal rate at any time since birth?	radio<br>1 Yes<br>0 No<br>2 Don't know
373	psq16_12	Is your child overweight?	radio<br>1 Yes<br>0 No<br>2 Don't know
374	psq17_12	Does your child often not seem to listen when spoken to directly?	radio<br>1 Yes<br>0 No<br>2 Don't know
375	psq18_12	Does your child often have difficulty organizing tasks and activities?	radio<br>1 Yes<br>0 No<br>2 Don't know
376	psq19_12	Is your child often easily distracted by extraneous stimuli?	radio<br>1 Yes<br>0 No<br>2 Don't know
377	psq20_12	Does your child fidget with his/her hands or feet or squirms in his/her seat?	radio<br>1 Yes<br>0 No<br>2 Don't know
378	psq21_12	Is your child 'on the go' or often act as if 'driven by a motor'?	radio<br>1 Yes<br>0 No<br>2 Don't know
379	psq22_12	Does your child often interrupt or intrude on others (e.g. butts into conversations or games)?	radio<br>1 Yes<br>0 No<br>2 Don't know

380	canker_sores_or_ulcers12	Section Header: *Other Symptoms*<br>Has your child had canker sores or ulcers in his/her mouth in the last 12 months?	radio<br>1 Yes<br>0 No<br>2 Don't know
381	ear_infxn12	How many ear infections has your child had in the last 12 months?	text
382	dysphagia12	Does your child ever have difficulty swallowing?	yesno<br>1 Yes<br>0 No
383	dysphagia_as_sym12<br>Show the field ONLY if:<br>[dysphagia12] = '1'	Is your child's difficulty swallowing related to any of the following symptoms? Please check all that apply.	checkbox<br>1 dysphagia_as_sym12___1 Fever<br>2 dysphagia_as_sym12___2 Tonsillitis<br>3 dysphagia_as_sym12___3 Canker sores or mouth ulcers<br>4 dysphagia_as_sym12___4 Ear infection<br>5 dysphagia_as_sym12___5 Large tonsils<br>6 dysphagia_as_sym12___6 Don't know<br>7 dysphagia_as_sym12___7 Other
384	dysphagia_as_sym_oth12<br>Show the field ONLY if:<br>[dysphagia_as_sym12(7)]='1'	Describe other.	text
385	dysphonia12	Does your child ever have difficult speaking?	yesno<br>1 Yes<br>0 No
386	dysphonia_as_sym12<br>Show the field ONLY if:<br>[dysphonia12] = '1'	Is your child's difficulty speaking related to any of the following symptoms? Please check all that apply.	checkbox<br>1 dysphonia_as_sym12___1 Fever<br>2 dysphonia_as_sym12___2 Tonsillitis<br>3 dysphonia_as_sym12___3 Canker sores or mouth ulcers<br>4 dysphonia_as_sym12___4 Ear infection<br>5 dysphonia_as_sym12___5 Large tonsils<br>6 dysphonia_as_sym12___6 Don't know<br>7 dysphonia_as_sym12___7 Other
387	dysphonia_as_sym_oth12<br>Show the field ONLY if:<br>[dysphonia_as_sym12(7)]='1'	Describe other.	text
388	speech_tx12	Has your child received speech therapy in the last 12 months?	yesno<br>1 Yes<br>0 No
389	sdays_miss12	How many days of school has your child missed in the last 12 months?	text (integer)
390	sdays_miss_ton12	How many of these missed school days were due to tonsil problems?	text
391	num_abx12<br>Show the field ONLY if:<br>[ton_num12] >= 1	How many times has your child gotten a course of antibiotics in the last 12 months?	text
392	osa_18_sleep_disturbance12	Section Header: *Quality of Life (OSA-18)*<br>Sleep disturbance	descriptive

393	osa_18_1_12	During the past 4 weeks, how often has your child had loud snoring?	radio<br>1 None of the time (1)<br>2 Hardly any of the time (2)<br>3 A little of the time (3)<br>4 Some of the time (4)<br>5 A good bit of the time (5)<br>6 Most of the time (6)<br>7 All of the time (7)
394	osa_18_2_12	During the past 4 weeks, how often has your child had breath-holding spells or pauses in breathing at night?	radio<br>1 None of the time (1)<br>2 Hardly any of the time (2)<br>3 A little of the time (3)<br>4 Some of the time (4)<br>5 A good bit of the time (5)<br>6 Most of the time (6)<br>7 All of the time (7)
395	osa_18_3_12	During the past 4 weeks, how often has your child had choking or making gasping sounds while asleep?	radio<br>1 None of the time (1)<br>2 Hardly any of the time (2)<br>3 A little of the time (3)<br>4 Some of the time (4)<br>5 A good bit of the time (5)<br>6 Most of the time (6)<br>7 All of the time (7)
396	osa_18_4_12	During the past 4 weeks, how often has your child had restless sleep or frequent awakening?	radio<br>1 None of the time (1)<br>2 Hardly any of the time (2)<br>3 A little of the time (3)<br>4 Some of the time (4)<br>5 A good bit of the time (5)<br>6 Most of the time (6)<br>7 All of the time (7)
397	osa_18_physical_symptoms_12	Physical symptoms	descriptive
398	osa_18_5_12	During the past 4 weeks, how often has your child had mouth breathing because of nasal obstruction?	radio<br>1 None of the time (1)<br>2 Hardly any of the time (2)<br>3 A little of the time (3)<br>4 Some of the time (4)<br>5 A good bit of the time (5)<br>6 Most of the time (6)<br>7 All of the time (7)

399	osa_18_6_12	During the past 4 weeks, how often has your child had frequent colds or upper respiratory infections?	radio<br>1 None of the time (1)<br>2 Hardly any of the time (2)<br>3 A little of the time (3)<br>4 Some of the time (4)<br>5 A good bit of the time (5)<br>6 Most of the time (6)<br>7 All of the time (7)
400	osa_18_7_12	During the past 4 weeks, how often has your child had nasal discharge or runny nose?	radio<br>1 None of the time (1)<br>2 Hardly any of the time (2)<br>3 A little of the time (3)<br>4 Some of the time (4)<br>5 A good bit of the time (5)<br>6 Most of the time (6)<br>7 All of the time (7)
401	osa_18_8_12	During the past 4 weeks, how often has your child had difficulty swallowing?	radio<br>1 None of the time (1)<br>2 Hardly any of the time (2)<br>3 A little of the time (3)<br>4 Some of the time (4)<br>5 A good bit of the time (5)<br>6 Most of the time (6)<br>7 All of the time (7)
402	osa_18_emotional_symptoms_12	Emotional symptoms	descriptive
403	osa_18_9_12	During the past 4 weeks, how often has your child had mood swings or temper tantrums?	radio<br>1 None of the time (1)<br>2 Hardly any of the time (2)<br>3 A little of the time (3)<br>4 Some of the time (4)<br>5 A good bit of the time (5)<br>6 Most of the time (6)<br>7 All of the time (7)
404	osa_18_10_12	During the past 4 weeks, how often has your child had aggressive or hyperactive behavior?	radio<br>1 None of the time (1)<br>2 Hardly any of the time (2)<br>3 A little of the time (3)<br>4 Some of the time (4)<br>5 A good bit of the time (5)<br>6 Most of the time (6)<br>7 All of the time (7)

405	osa_18_11_12	During the past 4 weeks, how often has your child had discipline problems?	radio<br>1 None of the time (1)<br>2 Hardly any of the time (2)<br>3 A little of the time (3)<br>4 Some of the time (4)<br>5 A good bit of the time (5)<br>6 Most of the time (6)<br>7 All of the time (7)
406	osa_18_daytime_function_12	Daytime function	descriptive
407	osa_18_12_12	During the past 4 weeks, how often has your child had excessive daytime sleepiness?	radio<br>1 None of the time (1)<br>2 Hardly any of the time (2)<br>3 A little of the time (3)<br>4 Some of the time (4)<br>5 A good bit of the time (5)<br>6 Most of the time (6)<br>7 All of the time (7)
408	osa_18_13_12	During the past 4 weeks, how often has your child had poor attention span or concentration?	radio<br>1 None of the time (1)<br>2 Hardly any of the time (2)<br>3 A little of the time (3)<br>4 Some of the time (4)<br>5 A good bit of the time (5)<br>6 Most of the time (6)<br>7 All of the time (7)
409	osa_18_14_12	During the past 4 weeks, how often has your child had difficulty getting up in the morning?	radio<br>1 None of the time (1)<br>2 Hardly any of the time (2)<br>3 A little of the time (3)<br>4 Some of the time (4)<br>5 A good bit of the time (5)<br>6 Most of the time (6)<br>7 All of the time (7)
410	osa_18_caregiver_concerns_12	Caregiver concerns	descriptive
411	osa_18_15_12	During the past 4 weeks, how often have the problems above caused you to worry about your child's general health?	radio<br>1 None of the time (1)<br>2 Hardly any of the time (2)<br>3 A little of the time (3)<br>4 Some of the time (4)<br>5 A good bit of the time (5)<br>6 Most of the time (6)<br>7 All of the time (7)

412	osa_18_16_12	During the past 4 weeks, how often have the problems above created concern that your child is not getting enough air?	radio<br>1 None of the time (1)<br>2 Hardly any of the time (2)<br>3 A little of the time (3)<br>4 Some of the time (4)<br>5 A good bit of the time (5)<br>6 Most of the time (6)<br>7 All of the time (7)
413	osa_18_17_12	During the past 4 weeks, how often have the problems above interfered with your ability to perform daily activities?	radio<br>1 None of the time (1)<br>2 Hardly any of the time (2)<br>3 A little of the time (3)<br>4 Some of the time (4)<br>5 A good bit of the time (5)<br>6 Most of the time (6)<br>7 All of the time (7)
414	osa_18_18_12	During the past 4 weeks, how often have the problems above made you frustrated?	radio<br>1 None of the time (1)<br>2 Hardly any of the time (2)<br>3 A little of the time (3)<br>4 Some of the time (4)<br>5 A good bit of the time (5)<br>6 Most of the time (6)<br>7 All of the time (7)
415	sleep_study12	Section Header: *Sleep Study*<br>Has your child had a sleep study in the last 12 months?	yesno<br>1 Yes<br>0 No
416	ahi12<br>Show the field ONLY if: [sleep_study12] = '1'	What was the AHI (apnea-hypopnea index)? If you do not know, please leave blank.	text
417	spo2_nadir12<br>Show the field ONLY if: [sleep_study12] = '1'	What was the SpO2 nadir? If you do not know, please leave blank.	text
418	height12	Section Header: *Height and Weight*<br>What is your child's current height (in inches)? If you do not know, please leave blank.	text (number, Min: 0)
419	weight12	What is your child's current weight (in pounds)? If you do not know, please leave blank.	text (number)
420	date12	Section Header: *Date*<br>Date of Survey	text (date_mdy)
421	parent_fname	Section Header: *Information for payment cards*<br>What is your first name?	text, Identifier
422	parent_middleinitial	What is your middle initial?	text
423	parent_lname	What is your last name?	text, Identifier
424	parent_ssn	What is your social security number?<br><br>Vanderbilt University Medical Center requires this information for tax purposes. Unfortunately, Vanderbilt University Medical Center will be unable to mail you a payment card without this piece of information.	text (number), Identifier
425	address	Please enter your current mailing address. The payment card will be mailed to this address.	notes

426	month_follow_up_survey_88ee_complete	Section Header: *Form Status*<br>Complete?	dropdown<br>0 Incomplete<br>1 Unverified<br>2 Complete

Instrument: **24 Month Follow Up Survey** (month_follow_up_survey_7b25) Collapse

427	new_mh24	Section Header: *Medical History*<br>Has your child been diagnosed with any new medical conditions in the last 12 months?	yesno<br>1 Yes<br>0 No
428	info_mh24<br>Show the field ONLY if:<br>[new_mh24] = '1'	Please provide more detail about your child's new medical conditions.	notes
429	hosp24	Has your child been hospitalized in the last 12 months?	yesno<br>1 Yes<br>0 No
430	info_hosp24<br>Show the field ONLY if:<br>[hosp24] = '1'	Please provide more detail about your child's hospitalization(s).	notes
431	new_sh24	Has your child undergone any surgeries or procedures in the in the last 12 months?	yesno<br>1 Yes<br>0 No
432	info_sh24<br>Show the field ONLY if:<br>[new_sh24] = '1'	Please provide more detail about your child's surgeries and/or procedures.	notes
433	ton_num24	Section Header: *Recurrent tonsillitis*<br>In the last 12 months, how many throat or tonsil infections has your child had?	text (integer)
434	ton_wfev24<br>Show the field ONLY if:<br>[ton_num24] >= 1	For how many of these episodes did your child have fever of 101 degrees F or higher?	text
435	ton_wstrep_pos24<br>Show the field ONLY if:<br>[ton_num24] >= 1	For how many of these episodes did your child have positive testing for strep?	text
436	rec_ton24<br>Show the field ONLY if:<br>[ton_num24] >= 1	When was the most recent episode? Please provide the date if known, otherwise estimate.	text
437	ton_as_sym24<br>Show the field ONLY if:<br>[ton_num24] >= 1	What symptoms did your child have when he/she had a throat or tonsil infection? Check all that apply:	checkbox<br>1 ton_as_sym24___1 Sore throat<br>2 ton_as_sym24___2 Swollen lymph nodes in neck<br>3 ton_as_sym24___3 Mouth ulcers<br>4 ton_as_sym24___4 Red tonsils<br>5 ton_as_sym24___5 Pus/white spots on tonsils<br>6 ton_as_sym24___6 Headache<br>7 ton_as_sym24___7 Abdominal pain<br>8 ton_as_sym24___8 Muscle aches<br>9 ton_as_sym24___9 Other
438	oth_ton_as_sym24<br>Show the field ONLY if:<br>[ton_as_sym24(9)] = '1'	Please describe "other"	text

439	add_ton24<br>Show the field ONLY if:<br>[ton_num24] >= 1	Please provide any additional information on tonsillitis episodes.	notes
440	psq1_24	Section Header: *Pediatric Sleep Questionnaire*<br>When sleeping, does your child snore more than half the time?	radio<br>1 Yes<br>0 No<br>2 Don't know
441	psq2_24	When sleeping, does your child always snore?	radio<br>1 Yes<br>0 No<br>2 Don't know
442	psq3_24	When sleeping, does your child snore loudly?	radio<br>1 Yes<br>0 No<br>2 Don't know
443	psq4_24	When sleeping, does your child have "heavy" or loud breathing?	radio<br>1 Yes<br>0 No<br>2 Don't know
444	psq5_24	When sleeping, does your child have trouble breathing, or struggle to breathe?	radio<br>1 Yes<br>0 No<br>2 Don't know
445	psq6_24	Have you ever seen your child stop breathing during the night?	radio<br>1 Yes<br>0 No<br>2 Don't know
446	psq7_24	Does your child tend to breathe through the mouth during the day?	radio<br>1 Yes<br>0 No<br>2 Don't know
447	psq8_24	Does your child have a dry mouth on waking up in the morning?	radio<br>1 Yes<br>0 No<br>2 Don't know
448	psq9_24	Does your child occasionally wet the bed?	radio<br>1 Yes<br>0 No<br>2 Don't know
449	psq10_24	Does your child wake up feeling unrefreshed in the morning?	radio<br>1 Yes<br>0 No<br>2 Don't know
450	psq11_24	Does your child have a problem with sleepiness during the day?	radio<br>1 Yes<br>0 No<br>2 Don't know

451	psq12_24	Has a teacher or other supervisor commented that your child appears sleepy during the day?	radio<br>1 Yes<br>0 No<br>2 Don't know
452	psq13_24	Is it hard to wake your child up in the morning?	radio<br>1 Yes<br>0 No<br>2 Don't know
453	psq14_24	Does your child wake up with headaches in the morning?	radio<br>1 Yes<br>0 No<br>2 Don't know
454	psq15_24	Did your child stop growing at a normal rate at any time since birth?	radio<br>1 Yes<br>0 No<br>2 Don't know
455	psq16_24	Is your child overweight?	radio<br>1 Yes<br>0 No<br>2 Don't know
456	psq17_24	Does your child often not seem to listen when spoken to directly?	radio<br>1 Yes<br>0 No<br>2 Don't know
457	psq18_24	Does your child often have difficulty organizing tasks and activities?	radio<br>1 Yes<br>0 No<br>2 Don't know
458	psq19_24	Is your child often easily distracted by extraneous stimuli?	radio<br>1 Yes<br>0 No<br>2 Don't know
459	psq20_24	Does your child fidget with his/her hands or feet or squirms in his/her seat?	radio<br>1 Yes<br>0 No<br>2 Don't know
460	psq21_24	Is your child 'on the go' or often act as if 'driven by a motor'?	radio<br>1 Yes<br>0 No<br>2 Don't know
461	psq22_24	Does your child often interrupt or intrude on others (e.g. butts into conversations or games)?	radio<br>1 Yes<br>0 No<br>2 Don't know
462	canker_sores_or_ulcers24	Section Header: *Other Symptoms*<br>Has your child had canker sores or ulcers in his/her mouth in the last 12 months?	radio<br>1 Yes<br>0 No<br>2 Don't know

463	ear_infxn24	How many ear infections has your child had in the last 12 months?	text
464	dysphagia24	Does your child ever have difficulty swallowing?	yesno<br>1 Yes<br>0 No
465	dysphagia_as_sym24<br>Show the field ONLY if:<br>[dysphagia24] = '1'	Is your child's difficulty swallowing related to any of the following symptoms? Please check all that apply.	checkbox<br>1 dysphagia_as_sym24___1 Fever<br>2 dysphagia_as_sym24___2 Tonsillitis<br>3 dysphagia_as_sym24___3 Canker sores or mouth ulcers<br>4 dysphagia_as_sym24___4 Ear infection<br>5 dysphagia_as_sym24___5 Large tonsils<br>6 dysphagia_as_sym24___6 Don't know<br>7 dysphagia_as_sym24___7 Other
466	dysphagia_as_sym_oth24<br>Show the field ONLY if:<br>[dysphagia_as_sym24(7)]='1'	Describe other.	text
467	dysphonia24	Does your child ever have difficult speaking?	yesno<br>1 Yes<br>0 No
468	dysphonia_as_sym24<br>Show the field ONLY if:<br>[dysphonia24] = '1'	Is your child's difficulty speaking related to any of the following symptoms? Please check all that apply.	checkbox<br>1 dysphonia_as_sym24___1 Fever<br>2 dysphonia_as_sym24___2 Tonsillitis<br>3 dysphonia_as_sym24___3 Canker sores or mouth ulcers<br>4 dysphonia_as_sym24___4 Ear infection<br>5 dysphonia_as_sym24___5 Large tonsils<br>6 dysphonia_as_sym24___6 Don't know<br>7 dysphonia_as_sym24___7 Other
469	dysphonia_as_sym_oth24<br>Show the field ONLY if:<br>[dysphonia_as_sym24(7)]='1'	Describe other.	text
470	speech_tx24	Has your child received speech therapy in the last 12 months?	yesno<br>1 Yes<br>0 No
471	sdays_miss24	How many days of school has your child missed in the last 12 months	text (integer)
472	sdays_miss_ton24	How many of these missed school days were due to tonsil problems?	text
473	num_abx24<br>Show the field ONLY if:<br>[ton_num24] >= 1	How many times has your child gotten a course of antibiotics in the last 12 months?	text
474	osa_18_sleep_disturbance24	Section Header: *Quality of Life (OSA-18)*<br>Sleep disturbance	descriptive

475	osa_18_1_24	During the past 4 weeks, how often has your child had loud snoring?	radio<br>1 None of the time (1)<br>2 Hardly any of the time (2)<br>3 A little of the time (3)<br>4 Some of the time (4)<br>5 A good bit of the time (5)<br>6 Most of the time (6)<br>7 All of the time (7)
476	osa_18_2_24	During the past 4 weeks, how often has your child had breath-holding spells or pauses in breathing at night?	radio<br>1 None of the time (1)<br>2 Hardly any of the time (2)<br>3 A little of the time (3)<br>4 Some of the time (4)<br>5 A good bit of the time (5)<br>6 Most of the time (6)<br>7 All of the time (7)
477	osa_18_3_24	During the past 4 weeks, how often has your child had choking or making gasping sounds while asleep?	radio<br>1 None of the time (1)<br>2 Hardly any of the time (2)<br>3 A little of the time (3)<br>4 Some of the time (4)<br>5 A good bit of the time (5)<br>6 Most of the time (6)<br>7 All of the time (7)
478	osa_18_4_24	During the past 4 weeks, how often has your child had restless sleep or frequent awakening?	radio<br>1 None of the time (1)<br>2 Hardly any of the time (2)<br>3 A little of the time (3)<br>4 Some of the time (4)<br>5 A good bit of the time (5)<br>6 Most of the time (6)<br>7 All of the time (7)
479	osa_18_physical_symptoms_24	Physical symptoms	descriptive
480	osa_18_5_24	During the past 4 weeks, how often has your child had mouth breathing because of nasal obstruction?	radio<br>1 None of the time (1)<br>2 Hardly any of the time (2)<br>3 A little of the time (3)<br>4 Some of the time (4)<br>5 A good bit of the time (5)<br>6 Most of the time (6)<br>7 All of the time (7)

481	osa_18_6_24	During the past 4 weeks, how often has your child had frequent colds or upper respiratory infections?	radio<br>1 None of the time (1)<br>2 Hardly any of the time (2)<br>3 A little of the time (3)<br>4 Some of the time (4)<br>5 A good bit of the time (5)<br>6 Most of the time (6)<br>7 All of the time (7)
482	osa_18_7_24	During the past 4 weeks, how often has your child had nasal discharge or runny nose?	radio<br>1 None of the time (1)<br>2 Hardly any of the time (2)<br>3 A little of the time (3)<br>4 Some of the time (4)<br>5 A good bit of the time (5)<br>6 Most of the time (6)<br>7 All of the time (7)
483	osa_18_8_24	During the past 4 weeks, how often has your child had difficulty swallowing?	radio<br>1 None of the time (1)<br>2 Hardly any of the time (2)<br>3 A little of the time (3)<br>4 Some of the time (4)<br>5 A good bit of the time (5)<br>6 Most of the time (6)<br>7 All of the time (7)
484	osa_18_emotional_symptoms_24	Emotional symptoms	descriptive
485	osa_18_9_24	During the past 4 weeks, how often has your child had mood swings or temper tantrums?	radio<br>1 None of the time (1)<br>2 Hardly any of the time (2)<br>3 A little of the time (3)<br>4 Some of the time (4)<br>5 A good bit of the time (5)<br>6 Most of the time (6)<br>7 All of the time (7)
486	osa_18_10_24	During the past 4 weeks, how often has your child had aggressive or hyperactive behavior?	radio<br>1 None of the time (1)<br>2 Hardly any of the time (2)<br>3 A little of the time (3)<br>4 Some of the time (4)<br>5 A good bit of the time (5)<br>6 Most of the time (6)<br>7 All of the time (7)

487	osa_18_11_24	During the past 4 weeks, how often has your child had discipline problems?	radio<br>1 None of the time (1)<br>2 Hardly any of the time (2)<br>3 A little of the time (3)<br>4 Some of the time (4)<br>5 A good bit of the time (5)<br>6 Most of the time (6)<br>7 All of the time (7)
488	osa_18_daytime_function_24	Daytime function	descriptive
489	osa_18_12_24	During the past 4 weeks, how often has your child had excessive daytime sleepiness?	radio<br>1 None of the time (1)<br>2 Hardly any of the time (2)<br>3 A little of the time (3)<br>4 Some of the time (4)<br>5 A good bit of the time (5)<br>6 Most of the time (6)<br>7 All of the time (7)
490	osa_18_13_24	During the past 4 weeks, how often has your child had poor attention span or concentration?	radio<br>1 None of the time (1)<br>2 Hardly any of the time (2)<br>3 A little of the time (3)<br>4 Some of the time (4)<br>5 A good bit of the time (5)<br>6 Most of the time (6)<br>7 All of the time (7)
491	osa_18_14_24	During the past 4 weeks, how often has your child had difficulty getting up in the morning?	radio<br>1 None of the time (1)<br>2 Hardly any of the time (2)<br>3 A little of the time (3)<br>4 Some of the time (4)<br>5 A good bit of the time (5)<br>6 Most of the time (6)<br>7 All of the time (7)
492	osa_18_caregiver_concerns_24	Caregiver concerns	descriptive
493	osa_18_15_24	During the past 4 weeks, how often have the problems above caused you to worry about your child's general health?	radio<br>1 None of the time (1)<br>2 Hardly any of the time (2)<br>3 A little of the time (3)<br>4 Some of the time (4)<br>5 A good bit of the time (5)<br>6 Most of the time (6)<br>7 All of the time (7)

494	osa_18_16_24	During the past 4 weeks, how often have the problems above created concern that your child is not getting enough air?	radio<br>1 None of the time (1)<br>2 Hardly any of the time (2)<br>3 A little of the time (3)<br>4 Some of the time (4)<br>5 A good bit of the time (5)<br>6 Most of the time (6)<br>7 All of the time (7)
495	osa_18_17_24	During the past 4 weeks, how often have the problems above interfered with your ability to perform daily activities?	radio<br>1 None of the time (1)<br>2 Hardly any of the time (2)<br>3 A little of the time (3)<br>4 Some of the time (4)<br>5 A good bit of the time (5)<br>6 Most of the time (6)<br>7 All of the time (7)
496	osa_18_18_24	During the past 4 weeks, how often have the problems above made you frustrated?	radio<br>1 None of the time (1)<br>2 Hardly any of the time (2)<br>3 A little of the time (3)<br>4 Some of the time (4)<br>5 A good bit of the time (5)<br>6 Most of the time (6)<br>7 All of the time (7)
497	sleep_study24	Section Header: *Sleep Study*<br>Has your child had a sleep study in the last 12 months?	yesno<br>1 Yes<br>0 No
498	ahi24<br>Show the field ONLY if:<br>[sleep_study24] = '1'	What was the AHI (apnea-hypopnea index)? If you do not know, please leave blank.	text
499	rdi24<br>Show the field ONLY if:<br>[sleep_study24] = '1'	What was the RDI (respiratory disturbance index)? If you do not know, please leave blank.	text
500	spo2_nadir24<br>Show the field ONLY if:<br>[sleep_study24] = '1'	What was the SpO2 nadir? If you do not know, please leave blank.	text
501	height24	Section Header: *Height and Weight*<br>What is your child's current height (in inches)? If you do not know, please leave blank.	text (number, Min: 0)
502	weight24	What is your child's current weight (in lbs)? If you do not know, please leave blank.	text (number)
503	date24	Section Header: *Date*<br>Date of Survey	text (date_mdy)
504	month_follow_up_survey_7b25_complete	Section Header: *Form Status*<br>Complete?	dropdown<br>0 Incomplete<br>1 Unverified<br>2 Complete
